# Effects of Indole-3-Acetic Acid on the Transcriptional Activities and Stress Tolerance of *Bradyrhizobium japonicum*

**DOI:** 10.1371/journal.pone.0076559

**Published:** 2013-10-02

**Authors:** Andrew J. Donati, Hae-In Lee, Johan H. J. Leveau, Woo-Suk Chang

**Affiliations:** 1 Department of Biology, University of Texas, Arlington, Texas, United States of America; 2 Department of Plant Pathology, University of California Davis, Davis, California, United States of America; Purdue University, United States of America

## Abstract

A genome-wide transcriptional profile of *Bradyrhizobium japonicum*, the nitrogen-fixing endosymbiont of the soybean plant, revealed differential expression of approximately 15% of the genome after a 1 mM treatment with the phytohormone indole-3-acetic acid (IAA). A total of 1,323 genes were differentially expressed (619 up-regulated and 704 down-regulated) at a two-fold cut off with *q* value ≤ 0.05. General stress response genes were induced, such as those involved in response to heat, cold, oxidative, osmotic, and desiccation stresses and in exopolysaccharide (EPS) biosynthesis. This suggests that IAA is effective in activating a generalized stress response in *B. japonicum*. The transcriptional data were corroborated by the finding that stress tolerance of *B. japonicum* in cell viability assays was enhanced when pre-treated with 1 mM IAA compared to controls. The IAA treatment also stimulated biofilm formation and EPS production by *B. japonicum*, especially acidic sugar components in the total EPS. The IAA pre-treatment did not influence the nodulation ability of *B. japonicum*. The data provide a comprehensive overview of the potential transcriptional responses of the symbiotic bacterium when exposed to the ubiquitous hormone of its plant host.

## Introduction

Indole-3-acetic acid (IAA) is an auxin-type phytohormone which plays a significant role in regulating cellular development in plants. It affects cellular elongation, differentiation, cellular division, apoptosis, and morphogenesis [1–3]. Auxins are capable of influencing gene expression in a highly efficient and selective manner by means of both positive and negative regulation [2].

Soil bacteria that reside in close association with plants, including pathogens, commensals, and endosymbionts, such as *Agrobacterium tumefaciens, Azospirillum brasilense, Pseudomonas syringae*, and *Bradyrhizobium japonicum* have been demonstrated to produce phytohormones such as IAA. This property is presumed to be a mechanism for altering the host plant’s physiology to the benefit of the bacteria [4–8]. *B. japonicum* is a bacterium of interest because it can exist in either a free-living state or an endosymbiont state in which it establishes a symbiotic relationship with the soybean plant (*Glycine max*) by engaging in specific signaling and a communication cascade which involves plant-borne flavonoids and bacterial lipo-chito-oligosaccharide Nod factors [9,10]. The ultimate result of the Nod factor-involved cascade on the soybean plant is organogenesis of root nodules in which bacteroids, an altered state of bacteria, reduce atmospheric nitrogen to ammonia for the benefit of the host plant. During the transition of the bacterium from a free-living state to a bacteroid state, the plant is concomitantly experiencing a localized structural and physiological change, which is mediated in part by hormones such as IAA.

*B. japonicum* has been shown to produce IAA at low concentrations either in the form of bacteroids within the soybean nodule [11] or under free-living conditions [12]. In addition to the synthesis of IAA, *B. japonicum* can also utilize exogenous IAA as a carbon source [13,14]. Studies of metabolite profiles by Egebo et al. indicate that the IAA degradation is an oxygen-dependent process [13]. Jensen et al. further identified intermediate compounds that illustrate a novel catabolic pathway for the IAA degradation in *B. japonicum* [14]. Little is known about how *B. japonicum* responds to exogenous IAA at the transcriptional level. To date, transcriptional profiles of bacteria in response to IAA are available for *Escherichia coli* [15,16], *A. tumefaciens* [17], *A. brasilense* [7], and *Sinorhizobium meliloti* [18]. However, none of them is an IAA degrader, although they are able to synthesize IAA. Thus, a comprehensive gene expression profile of *B. japonicum* would be of considerable importance not only for identifying genes required for IAA catabolism as a symbiotic nitrogen fixer, but also for making inferences on the biological relevance of the phytohormone in the symbiosis with its host plant. Here we propose that the transcriptional responses and the associated physiological changes will confer some fitness advantage to *B. japonicum*’s ability to survive in the rhizosphere that often undergoes fluctuations of environmental factors such as temperature, osmotic pressure, reactive oxygen species, and desiccation.

## Materials and Methods

### Bacterial strains and culture conditions

*B. japonicum* USDA 110 was cultured in arabinose-gluconate (AG) medium which was comprised of 125 mg NaHPO_4_, 250 mg Na _2_SO_4_, 320 mg NH_4_Cl, 180 mg MgSO_4_·7H_2_O, 10 mg CaCl_2_, 4 mg FeCl_3_, 1.3 g HEPES, 1.1 g MES, 1.0 g yeast extract, 1.0g L-arabinose, and 1.0 g D-gluconic acid sodium salt per liter with pH adjusted to 6.8 [19]. Cultures were maintained with full aeration at 30 °C with shaking (200 rpm).

### Growth conditions for IAA treatments

The effects of IAA on the growth and survival characteristics of *B. japonicum* were investigated. Briefly, cells were grown in a 1-liter-flask containing 350 ml of the AG medium until mid-log phase in order to capture the cells in a metabolically active state (O.D._600_ ~ 0.8 to 1.0). The culture was divided into six 50-ml cultures by pipetting into 250-ml flasks and each culture was subsequently treated with a range of IAA concentrations (0, 0.25, 0.5, 1, 2, and 5 mM). For the 0 mM IAA control treatment, the same volume of ethanol was added, since ethanol was used as a solvent to dissolve IAA. After IAA treatments, measurements of turbidity (O.D._600_) were taken at 4, 16, and 28 h after treatment with a UV-Vis spectrophotometer (Genesys 5, Spectronic Instruments) and plotted against time.

### Detection and quantification of IAA *in vitro*

The effective concentration of IAA in the cultures was detected and quantified by the use of the Salkowski staining reagent [20]. The reagent was prepared by slowly dissolving 2.4 g FeCl_3_ in a solvent comprised of 88 ml H_2_SO_4_ and 100 ml H_2_O. The reagent was subsequently stored in the dark. IAA in the culture was quantified by mixing 0.5 ml of the Salkowski reagent with 0.5 mL of the sample and incubating the samples in the dark for 1 h. The absorbance at 540 nm was measured and the measurements were compared to a standard curve to infer effective concentration of IAA in the culture. IAA was also added to cell-free media for each concentration to rule out a possible effect of the medium on IAA biodegradation. Three replicates were included for all measurements.

### RNA isolation

The IAA-treated cells were harvested from 100-ml cultures during mid-log phase (O.D._600_ ~0.8-1.0) after treatment with 1 mM IAA (0.2% v/v) for 3 h by the addition of 10% stop solution (5% H_2_O-phenol, pH 4.3, in 100% ethanol). The control cells were also harvested form 100-ml cultures treated with solvent (ethanol, 0.2% v/v) for 3 h by the addition of 10% stop solution. Cells were recovered by centrifugation at 8,000 x *g* for 20 min, flash frozen in liquid N_2_, and stored at -80 °C until use. Total RNA was isolated from cell pellets using a modified hot-phenol method as previously described [21]. DNase treatment and purification of total RNA were performed by using RNase-free DNase set (Qiagen) and the RNeasy® mini kit (Qiagen) according to the manufacturer’s protocols. RNA quality was analyzed on 0.8% agarose gels and RNA quantity was measured by the NanoDrop ND-1000 spectrophotometer (Thermo Scientific).

### Analysis of genome-wide transcriptional activities by microarray hybridization

Genome-wide transcriptional profiles were generated from the hybridization of cDNA samples labeled with Amersham Cy3 and Cy5 monoreactive dyes (GE Healthcare) to microarray chips containing 70-mer oligonucleotides which were complementary to each of the 8,453 annotated open reading frames (ORFs) of *B. japonicum* [22]. Thirty micrograms of input total RNA were used for cDNA synthesis and 5 µg of cDNA from both control and experimental conditions were used for labeling and hybridization. The detailed protocols for cDNA synthesis, cDNA labeling, hybridization, and washing have been described [22]. The hybridizations were performed with three independent biological replicates for each condition, as well as a dye-swap for each replicate, resulting in a total of 6 slides as was previously described [22].

### Statistical analysis of microarray data

The slides were scanned with the Axon GenePix 4200 scanner and GenePix Pro 6.0 software was used to measure intensity values at each spot. The Lowess spatial smoothing algorithm and mixed-effect microarray ANOVA (MAANOVA) were used to standardize the signal intensities to account for slide and spot abnormalities [23]. The significance analysis of microarray (SAM) statistical package [24] was used to create a list of differentially expressed genes with a fold-induction threshold of 2.0, and a false-discovery rate (FDR) of 5% or less (*q* value ≤ 0.05). The microarray data from this study are compiled in the NCBI Gene Expression Omnibus (GEO) database (http://www.ncbi.nlm.nih.gov/geo/) and are accessible through the GEO series accession numbers GSE36913.

### Quantitative reverse transcription-PCR (qRT-PCR) analysis

For the purpose of confirmation, representative genes which were differentially expressed from the microarray data were chosen for qRT-PCR analysis. Primers were designed with Primer 3 software, available at http://frodo.wi.mit.edu/primer3/, to amplify 80-250 bp regions of the chosen genes (Table S1). The source of the input RNA was the same as that used in the microarray experiment. The process of cDNA synthesis and qRT-PCR was performed according to a previously described protocol [25]. Melting curve analysis was performed on Sequence Detection System software (version 1.3; Applied Biosystems) to check the specificity of PCR products and the LinRegPCR software was used to determine the target mRNA quantity (R_0_) and amplification efficiency (E) (Table S2) [26]. Relative expression values from three technical replicates of each biological replicate (a total of three biological replicates) were normalized to the expression values of a housekeeping gene (bll0631, *par*A) [25], which encodes a chromosome partitioning protein. This gene was constitutively expressed under both IAA treatment and control conditions. Fold-induction values were calculated in accordance with a previously described method [27].

### Stress tolerance tests

The effects of a 1 mM IAA pre-treatment on tolerance to oxidative stress, heat shock, cold shock, osmotic stress, and desiccation stress were examined. Cultures were grown to mid-log phase (O.D._600_ ~ 1.0) and then divided evenly for all tests. One secondary culture from each replicate received a 1 mM IAA treatment for 3 h, while the other did not (control). Both secondary cultures were subsequently exposed to the exogenous stress variable. The experimental conditions were as follows: heat shock (42 °C or 50 °C for 10 min.); cold shock (4 °C for 4, 24, and 72 h); osmotic stress (0.5M NaCl for 4 h), desiccation stress (27% relative humidity [RH] for 72 h), and oxidative stress (10 mM H2O2 for 20 min). The osmotic, desiccation, and oxidative conditions were chosen based on our previous studies [22,25,28]. Samples were taken at the indicated time points and serially diluted before being spread on AG agar plates. CFUs were counted after 3-5 days of incubation at 30 °C and recorded for each replicate. Three independent replicates were included for each condition and time variable. Percent survival was calculated by using the viable cell count at t = 0 as a reference for each sample set.

### Exopolysaccharide (EPS) quantification

The effect of 1 mM IAA on the EPS production phenotype was assayed. The mid-log phase cultures treated with or without 1 mM IAA were grown to stationary phase and then harvested by centrifugation at 16,000 × g for 30 min at 4 °C. The supernatant containing extracellular materials was filtered (pore size, 0.45 µm) and treated with DNase I and proteinase K as described previously [29,30]. EPS was precipitated overnight with 3 volumes of ethanol at -20 °C. A second ethanol precipitation was performed to ensure adequate EPS recovery [31,32]. EPS was dried at room temperature before being resuspended in deionized water. Total carbohydrate content was measured by the phenol-sulfuric acid method [33] with glucose (Glc) as the standard, and the acidic carbohydrate content was measured by the *m-*phenylphenol method [34] with D-glucuronic acid (GlcUA) as the standard. Total protein content was also measured by the Bradford’s method [35] to normalize the carbohydrate content to the total protein. Six independent replicates were included for each condition.

### Biofilm assay

A quantitative biofilm assay was performed in polystyrene 96-well microtiter plates as described previously [36,37]. Briefly, *B. japonicum* cells were harvested from 10 ml of mid-log phase culture by centrifugation at 8,000 × g for 5 min. The cell pellet was washed and resuspended in Bergersen minimal medium [38] with 0.4% glycerol (BMM) to adjust to an OD_600_ of 1. The cell suspension was 0.5% (vol/vol) diluted (initial OD_600_ was 0.005; ca 5 × 10^6^ cells/ml) into BMM supplemented with different concentrations of IAA (final concentrations were 0.1, 0.25, 0.5, and 1 mM, respectively), or its solvent ethanol. The same volume (0.2% vol/vol) of ethanol, but different concentrations of IAA, was added to each treatment. One hundred fifty microliter volumes were transferred into the wells (6 wells per sample) of polystyrene 96-well plates. The plates were then incubated at 30 °C without shaking. Specific biofilm formation (SBF) was calculated as follows [39]: SBF = (B-C)/G, where B is the OD_595_ of the attached biofilm cells, C is the OD_595_ of the stained control wells containing the medium only, and G is the OD_600_ of cells grown in broth. Experiments were independently repeated three times.

### Nodulation assay

Soybean seeds for the nodulation assay were surface-sterilized and germinated as described previously [37]. The sterilized seeds were transferred into plastic plant growth pouches (Mega International) and inoculated with 1 ml of the cell suspension (OD_600_ = 0.1; ca. 1 × 10^8^ CFU/ml) of *B. japonicum* pretreated with various concentrations of IAA for 3 h. The inoculated seedlings were grown in a plant growth chamber at 26 °C with 16 h of day and 8 h of night. After 21 days post inoculation, the number of nodules and the distance from the initial root tip were determined as described previously [40]. Subsequently, nodules were detached from the roots using sterilized fine point tweezers. The detached nodules and the whole plants without nodules were dried in an oven at 70°C for 3 days, and the dry weights of both were measured. A total of 9 plants were used for each treatment in the nodulation assay and all experiments were repeated three times.

## Results

### IAA affects *B. japonicum* growth and survival

**Figure 1:**
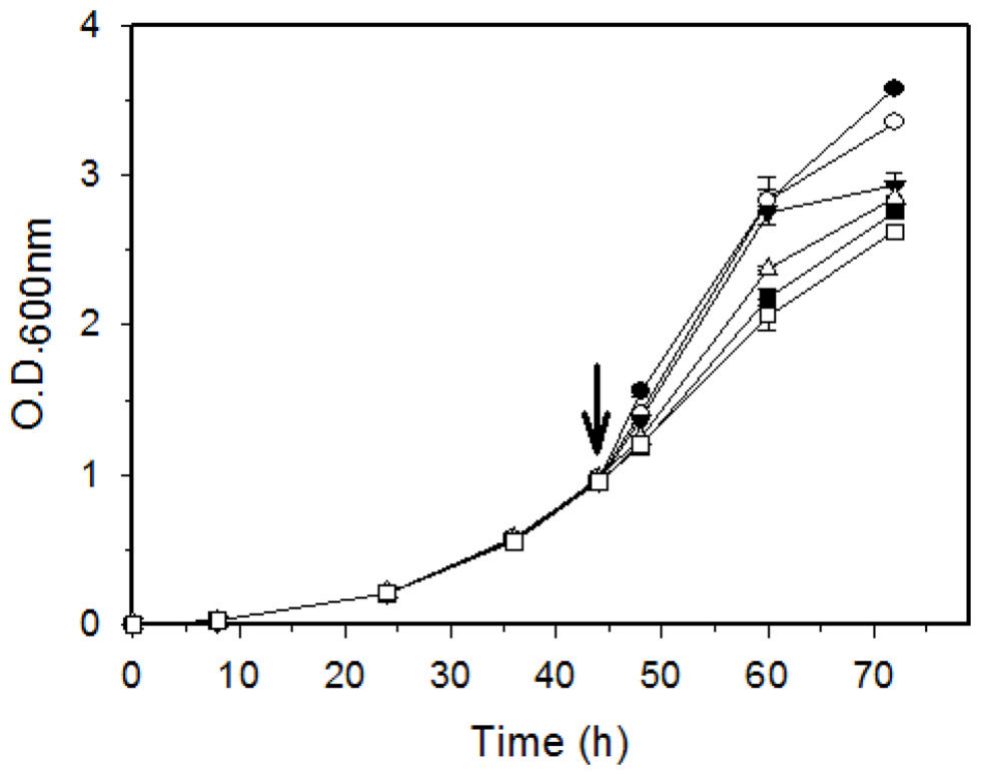
The growth characteristics of *B. japonicum* in response to IAA treatments. An arrow indicates the time (t = 44 h; O.D._600_ ~ 1.0) at which IAA was added to cultures. The symbol legend is as follows: (-●-), control (no IAA); (-○-), 0.25 mM; (--), 0.5 mM; (-Δ-), 1 mM; (-■-), 2 mM; and (-□-), 5 mM. Each point is the mean ± standard error of the mean for three biological replicates.

**Figure 2:**
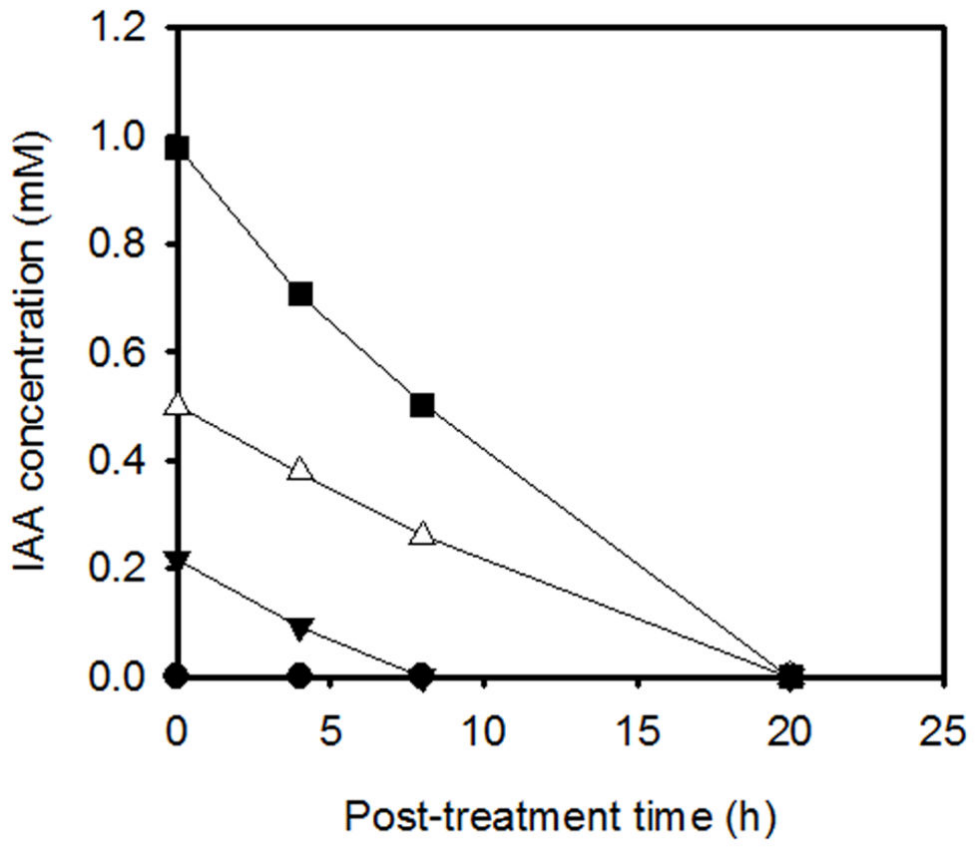
The concentration of IAA in the supernatant plotted as a function of time. Time on the X-axis corresponds to post-treatment time. The symbol legend is as follows: (-●-), control (no IAA); (--), 0.25 mM; (-Δ-), 0.5 mM; and (-■-), 1 mM. Each point is the mean ± standard error of the mean for two independent experiments, each comprising three replications. No error bar is shown due to too small variances.

We investigated the effects of the phytohormone IAA on the growth and survival of *B. japonicum* across a range of concentrations (0.25, 0.5, 1, 2, and 5 mM) during mid-log to early stationary phase. After the addition of IAA to a culture in mid-exponential phase (O.D._600_ ~ 1.0), the growth rate appeared to decrease in a concentration-dependent manner, but there was no statistically significant reduction in optical cell densities up to 0.5 mM at 16 h after exposure to IAA (Figure 1). Significant reduction was observed in 1, 2, and 5 mM treatments compared to the control treatment at 16 and 28 h after exposure to IAA. We selected the 1 mM concentration for the transcriptomics study and further experiments, since it showed an intermediate effect on the growth of *B. japonicum* cells. The same concentration was also used in other studies investigating the impact of IAA on transcription and phenotype [7,41]. Additionally, we monitored the disappearance of the added IAA, because *B. japonicum* is capable of IAA degradation. Within 8 h, all IAA had disappeared in cultures with 0.25 mM IAA (Figure 2). IAA was no longer detectable after 20 h for the 0.5 and 1 mM conditions. For the 1 mM condition after 3 h, approximately 0.8 mM or 80% of the treatment was still detectable in the medium (Figure 2). However, the remaining cultures might contain oxidized or conjugated forms of IAA besides free IAA because the Salkowski method could detect all kinds of IAA derivatives. The levels of IAA stayed constant for the cell-free medium (data not shown), which eliminates a possible effect of the medium itself on IAA degradation.

### The genome-wide transcriptional profile reveals that IAA has a significant effect on *B. japonicum* gene expression

**Figure 3:**
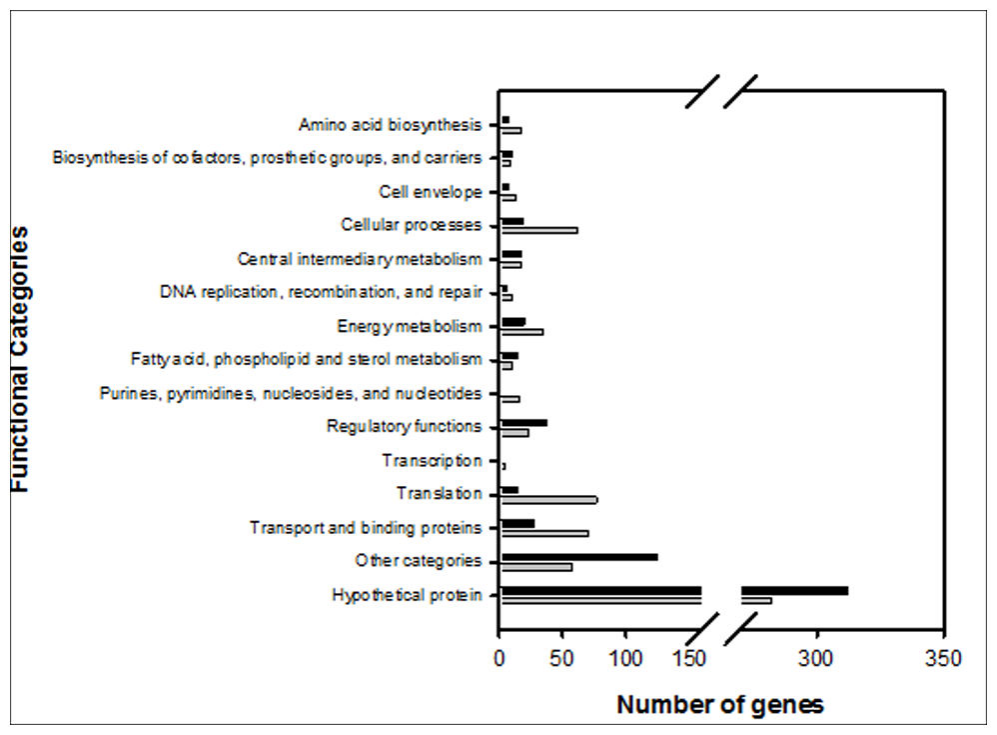
Functional classification of the genes differentially expressed after IAA treatment. The cells harvested from mid-log phase cultures were treated with 1 mM IAA for 3 h. The microarray results were analyzed with a 2.0-fold cut off and *q* value ≤ 0.05. Black bars represent positive fold-induction values and grey bars represent negative fold-induction values. Functional classifications were derived from *B. japonicum* genome annotations available through Rhizobase (http://bacteria.kazusa.or.jp/rhizobase/).

**Figure 4:**
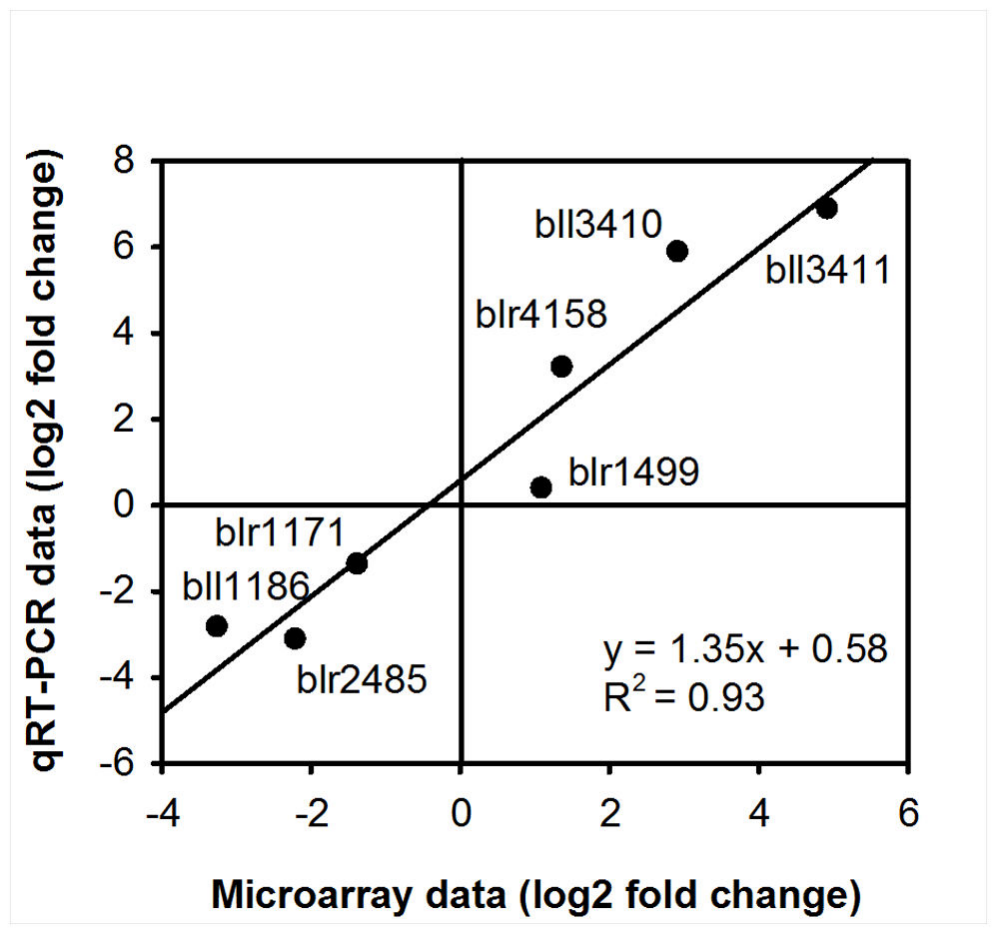
Comparison of log_2_ transformed qRT-PCR data and microarray data. Seven representative genes were selected based on fold-induction and functional categories. The corresponding gene names for the locus IDs are as follows: bll3410 (iorB); bll3411 (iorA); blr1499 (exoN); blr1171 (coxA); bll1186 (*atpB*'). blr 2485 and blr4158 lack assigned names.

A total of 1,323 genes (619 up-regulated and 704 down-regulated) out of the 8,543 annotated open reading frames (15.5%) for *B. japonicum* exhibited differential expression in response to a 3 h exposure to 1 mM IAA with a 2.0 fold cut-off and *q* ≤ 0.05 (Table S3). Representatives from all the major functional group classifications are shown in Figure 3. The majority of differentially-expressed genes classified as involved in amino acid biosynthesis, cellular processes, nucleic acid-related functions, energy metabolism, translation, and transport and binding proteins were repressed. Many genes associated with regulatory functions and undefined or hypothetical functions were up-regulated. The expression levels of representative genes from various functional categories were determined by qRT-PCR analysis to confirm the microarray-based gene expression data. As shown in Figure 4, the results were consistent with the microarray data.

Energy metabolism related genes in general were repressed (Table 1). Both putative ATP-synthase operons were down-regulated along with proteins associated with the ubiquinol-cytochrome c complex (complex III) in the aerobic respiratory chain such as a Rieske iron-sulfur protein (blr2485) and a cytochrome b/c1 precursor (blr2486). Although most of the genes associated with energy metabolism were repressed, the two subunits of an indolepyruvate ferredoxin oxidoreductase encoded by bll3410 and bll3411 (Table 1) and a locus (blr4158) involved in the oxygen-dependent catabolism of IAA which presumably encodes a probable tryptophan 2,3-dioxygenase [13] were induced (Table S3). These genes are likely the most obvious candidates for the observed degradation of IAA by *B. japonicum*.

**Table 1:** A subset of the *B. japonicum* genes which were differentially expressed in response to a 1 mM IAA treatment for 3 h^**a**^.

Locus ID (Gene name)	Description^b^	Fold induction
Energy metabolism		
bll0439 (*atpC*)	ATP synthase epsilon chain	-3.02
bll0440 (*atpD*)	ATP synthase beta chain	-2.25
bll0441 (*atpG*)	ATP synthase gamma chain	-3.09
bll0442 (*atpA*)	ATP synthase alpha chain	-2.88
blr0573	acetyl-CoA synthetase	-2.23
blr1170 (*coxB*)	cytochrome C oxidase subunit II	-2.05
blr1171 (*coxA*)	cytochrome C oxidase subunit I	-2.54
blr1175 (*coxC*)	cytochrome C oxidase subunit III	-2.30
bll1185 (*atpB*)	FoF1 ATP synthase B chain	-7.52
bll1186 (*atpB*')	FoF1 ATP synthase B' chain	-9.46
bsl1187 (*atpC*)	FoF1 ATP synthase C chain	-8.59
bll1188 (*atpA*)	FoF1 ATP synthase A chain	-4.64
bsl1189 (*atpI*)	FoF1 ATP synthase subunit I	-3.86
blr1423 (*cycM*)	cytochrome c	-2.39
blr2485	rieske iron-sulfur protein	-4.55
blr2486	cytochrome b/c1 precursor	-3.89
blr7525 (*hisG*)	ATP phosphoribosyltransferase	-2.29
bll3410 (*iorB*)	indolepyruvate ferredoxin oxidoreductase beta subunit	7.64
bll3411 (*iotA*)	indolepyruvate ferredoxin oxidoreductase alpha subunit	30.70
bll7906	putative ferredoxin	2.35
Chaperones		
bll0729 (*hspH*)	small heat shock protein	3.92
blr1100 (*hsIO*)	heat shock protein 33	2.81
blr2450 (*htpx*)	protease heat shock protein	2.43
blr5220 (*hspE*)	small heat shock protein	3.13
blr5221 (*hspF*)	small heat shock protein	2.08
blr5226 (*groES1*)	heat shock protein	2.17
blr5227 (*groEL1*)	heat shock protein	2.39
blr5233 (*hspB*)	small heat shock protein	2.38
blr7740	small heat shock protein	2.21
bsl8249 (*cspA*)	cold shock protein	6.02
EPS formation		
blr1499 (*exoN*)	UTP-glucose-1-phosphate uridylyltransferase	2.16
blr2358	probable glycosyl transferase	3.47
bll2752	probable glycosyl transferase	2.33
blr7578 (*exoB*)	UDP-glucose 4'-epimerase	2.01
bll8163	glycosyl transferase	2.39
Oxidative stress		
blr3428	organic hydroperoxide resistance protein	17.10
bll4012	organic hydroperoxide resistance protein	2.34
DNA replication, recombination		
and repair		
bll0830	chromosomal replication initiator protein	-4.16
bll5755	RecA protein	-3.01
bll0827	DNA replication and repair protein	4.05
bll4072	replicative DNA helicase	10.60
Symbiosis		
blr1812 (*nolB*)	nodulation protein	-4.85
blr1814 (*nolU*)	nodulation protein	-2.57
blr1815 (*nolV*)	nodulation protein	-2.89
blr2025 (*nodA*)	acyl transferase	-4.78
bll1714	two component regulator	2.11
bll2067	nodulate formation efficiency C protein	5.60
bll2073	NoeE homolog	2.64
bll4952	NfeD protein homolog	2.29
Nitrogen fixation		
blr1063	putative autoinducer synthase	-4.59
blr2036 (*fixR*)	Oxidoreductase	-2.54
blr2763 (*fixN*)	cytochrome-c oxidase	-5.24
blr2764 (*fixO*)	cytochrome-c oxidase	-5.02
bsr2765 (*fixQ*)	*cbb3* oxidase subunit IV	-5.46
blr2766 (*fixP*)	*cbb3* oxidase subunit III	-3.03
blr2767 (*fixG*)	iron-sulfur cluster-binding protein	-3.08
blr2768 (*fixH*)	FixH protein	-3.90
blr3125 (*cycH*)	cytochrome C-type biogenesis protein	-2.01
bll1167 (*sipS*)	signal peptidase	2.29
blr1755	*R. etli iscN* homolog	2.30
blr1769 (*nifH*)	dinitrogenase reductase protein	3.08
blr1774 (*fixC*)	Flavoprotein	2.22
blr2037 (*nifA*)	*nif*-specific regulatory protein	6.85
blr2038 (*fixA*)	electron transfer flavoprotein beta chain	2.18
blr2694	VirG-like two component response regulator	9.20
blr3126 (*cycJ*)	cytochrome C-type biogenesis protein	3.11

a Differentially expressed genes from each functional category were selected based on a 2-fold cut-off with *q* value ≤ 0.05.

b Gene description (annotations) represents the third level from the three-tiered functional level system of *B. japonicum* (http://genome.kazusa.or.jp/rhizobase/Bradyrhizobium/genes/category).

Within cellular processes, a number of molecular chaperones including 9 heat-shock and 1 cold-shock proteins were up-regulated (Table 1). At least five EPS biosynthesis-related genes and several oxidative stress response genes such as organic hydroperoxide resistance proteins were also induced (Table 1). Several DNA replication, recombination and repair proteins were induced to high levels, such as a replicative DNA helicase (bll4072) and a DNA replication and repair protein (bll0827), although a gene encoding the RecA protein was repressed (Table 1).

The effects of IAA exposure on symbiosis and nitrogen fixation-related genes involved both induction and repression (Table 1). For example, blr1769 (*nifH*), which encodes a dinitrogenase reductase protein, and blr2037 (*nifA*), which encodes a *nif*-specific regulatory protein, were induced 3.08 and 6.85 fold, respectively. Members of the *fixNOQP* operon such as blr2763, blr2764, bsr2765 and blr2766 which encode proteins involved in an alternative, high-affinity cytochrome *c* (cbb3) oxidase complex in the respiratory chain [42,43] were repressed 5.24, 5.02, 5.46 and 3.03 fold, respectively. An iron-sulfur cluster-binding protein (blr2767) and a FixH protein (blr2768), which are members of the *fixGHIS* operon were also repressed 3.08 and 3.90 fold, respectively (Table 1).

### IAA pre-treatment promotes an enhanced stress tolerance phenotype and increased EPS production

The genome-wide gene expression data revealed the induction of several heat shock, cold shock, EPS biosynthetic and molecular chaperone proteins (Table 1), which are associated with a general stress response. To test whether IAA exposure makes *B. japonicum* cells more resistant to stress, we performed various cell viability assays in response to heat, cold, osmotic, oxidative, and desiccation stresses (Table 2). Cell cultures that received a 1 mM pretreatment with IAA for 3 h exhibited enhanced tolerance or decreased sensitivity to heat shock stress at 42 °C and 50 °C, cold shock at 4 °C with the exception of the 24 h time variable, exposure to 10 mM H_2_O_2_ for 20 min, as well as osmotic (0.5M NaCl for 4 h) and desiccation (27% RH for 72 h) stresses.

**Table 2:** Increased stress tolerance of *B. japonicum* after exposure to IAA.

Environmental Cues	Survival (%)^a^	
	(+) IAA	(-) IAA
Heat shock		
42 °C for 10 min	103.2 ± 6.0	60.8 ± 3.2*
50 °C for 10 min	84.8 ± 7.0	47.6 ± 2.5*
Cold Shock		
4 °C for 4 h	112.0 ± 2.1	72.5 ±3.4*
4 °C for 24 h	98.8 ± 8.9	72.6 ± 1.3
4 °C for 72 h	81.9 ± 10.2	62.8 ± 3.1*
Oxidative stress		
10 mM H_2_O_2_ for 20 min	96.3 ± 4.5	67.8 ± 5.6*
Osmotic stress		
0.5M NaCl for 4 h	86.4 ± 7.2	49.0 ± 1.3*
Desiccation stress		
27% RH for 72 h	89.3 ± 0.5	84.9 ± 0.1*

a % Survival is derived by the ratio of CFUs/mL after 1 mM IAA treatment over the CFUs/mL before the treatment. Values are the mean ± the standard error of the mean for three independent experiments with three replications each.

* An asterisk indicates that differences in this row of data are statistically significant with a *P* ≤ 0.05 using Student’s t-test.

**Figure 5:**
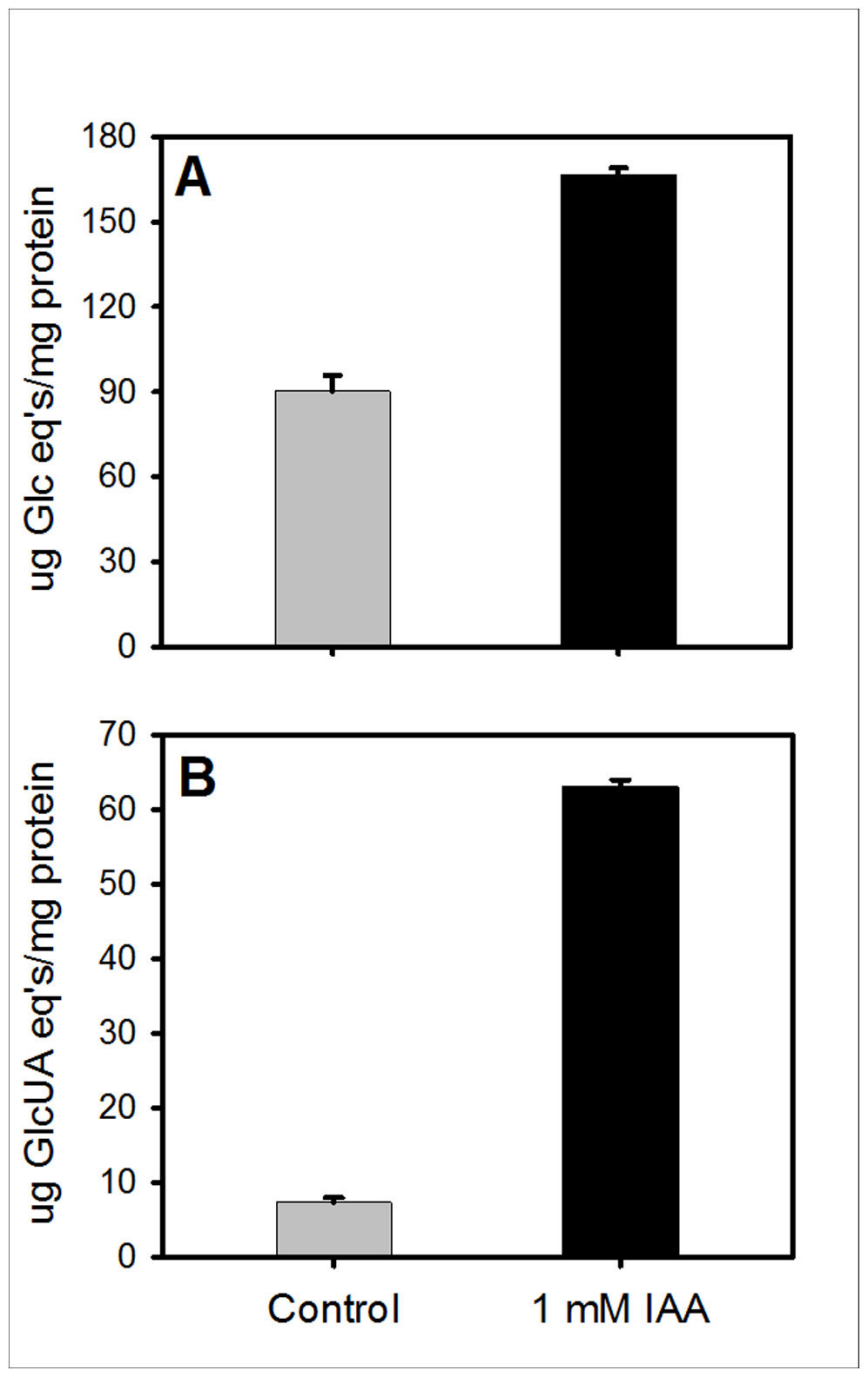
Quantification of the total EPS (A) and acidic carbohydrate content (B). EPSs were isolated from the supernatant of *B. japonicum* cultures treated with 1 mM IAA (black bars) or control (grey bars). The differences between the two are statistically significant (*P* < 0.05). Values are means ± standard errors of the means for three independent experiments with three replications each.

Total EPS production increased significantly in response to 1 mM IAA (Figure 5A). The EPSs are reported as total carbohydrate content (Figure 5A) and acidic carbohydrate content (Figure 5B). Specifically, the total sugar content was increased by ca. 2-fold and the acid sugar content by ca. 9-fold. The increase in the total sugar content was mainly due to the increase in acidic sugar residues in the EPS. This result indicates that the enhanced tolerant phenotype of the IAA pre-treated cells is correlated with more production of acidic EPS.

### IAA treatment also enhances biofilm formation

**Figure 6:**
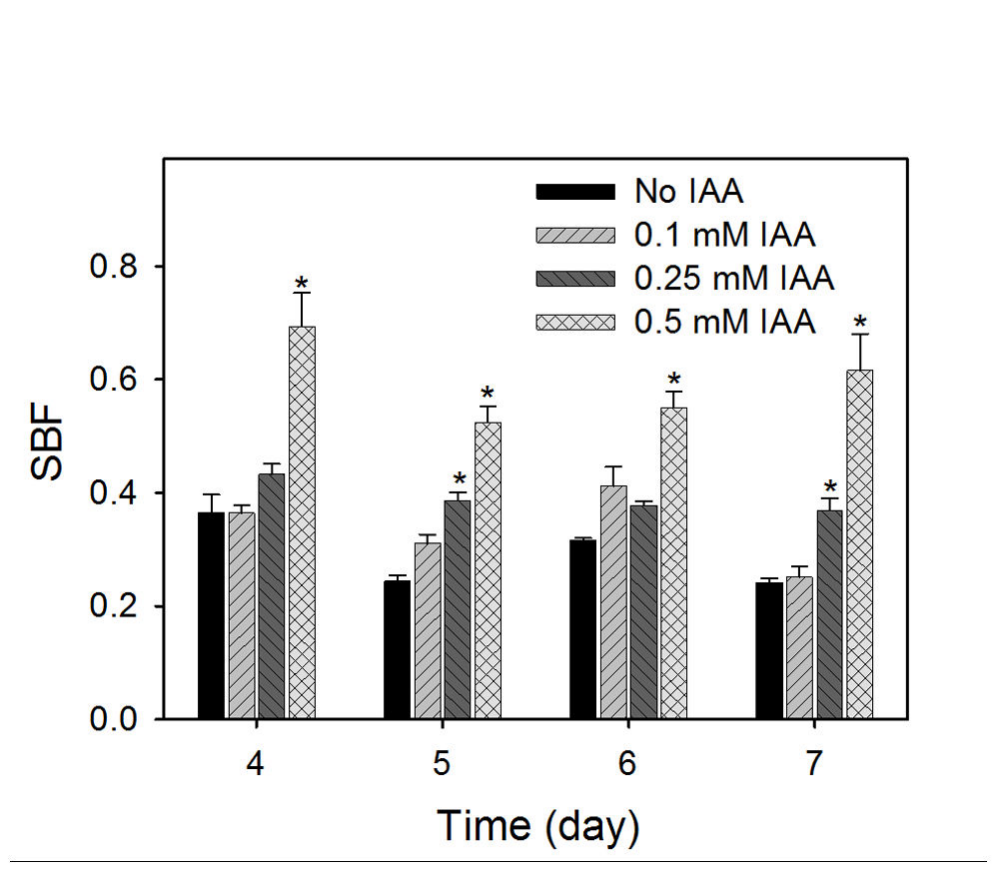
Effects of different concentrations of IAA on SBF by *B. japonicum* in minimal medium. The amount of biofilm was determined by the crystal violet staining method and calculated as follows: SBF = (B-C)/G, where B is the OD_595_ of the attached biofilm cells, C is the OD_595_ of the stained control wells containing the medium only, and G is the OD_600_ of cells grown in broth. Values are means ± standard errors of the means for three independent experiments with three replications each. An asterisk (*) indicates a significant difference between the treatment and the control (no IAA), with a *P* < 0.05 using Student’s t-test.

Since EPS production is a hallmark of biofilm-related resistance and its development, we monitored biofilm formation by IAA-treated *B. japonicum* cells by a crystal violet staining method. Biofilm-forming ability of the cells was expressed as specific biofilm formation (SBF), a quantitative biofilm formation unit normalized to cell density [39], and SBF was monitored from 4 to 7 days after inoculation. In general, biofilm formation was increased in a concentration-dependent manner, but there was no statistically significant difference of SBF between the 0.1 mM treatment and the control (no IAA) during the 4- to 7-day incubation (Figure 6). For the 0.25 mM treatment, there was also a general trend of enhanced biofilm formation compared to the control, but SBF at only 5 and 7 days after inoculation showed significant difference (*P* < 0.05). When *B. japonicum* was grown in 0.5 mM IAA, SBF was significantly higher (*P* < 0.05) than that of the control at all time points examined (ca. 1.7- to 2.5-fold increase at 4 to 7 days) (Figure 6). This result is analogous to a previous study that the biofilm formation of 0.5 mM IAA-treated *E. coli* cells was increased [15].

Interestingly, we were not able to observe biofilm development at 1 mM IAA, since there was no considerable growth in the biofilm experiment (data not shown). Compared to the survival study shown in Figure 1, lower cell density cultures were used to monitor biofilm formation: ca. 5 × 10^6^ cells/ml (O.D._600_ ~ 0.005) and ca. 1 × 10^9^ cells/ml (O.D._600_ ~ 1.0) were treated with 1 mM IAA for the biofilm experiment and survival study, respectively. This indicates cell mass-dependent growth and survival in response to the high concentration of IAA.

### IAA pre-treatment does not influence the nodulation ability of *B. japonicum*

**Figure 7:**
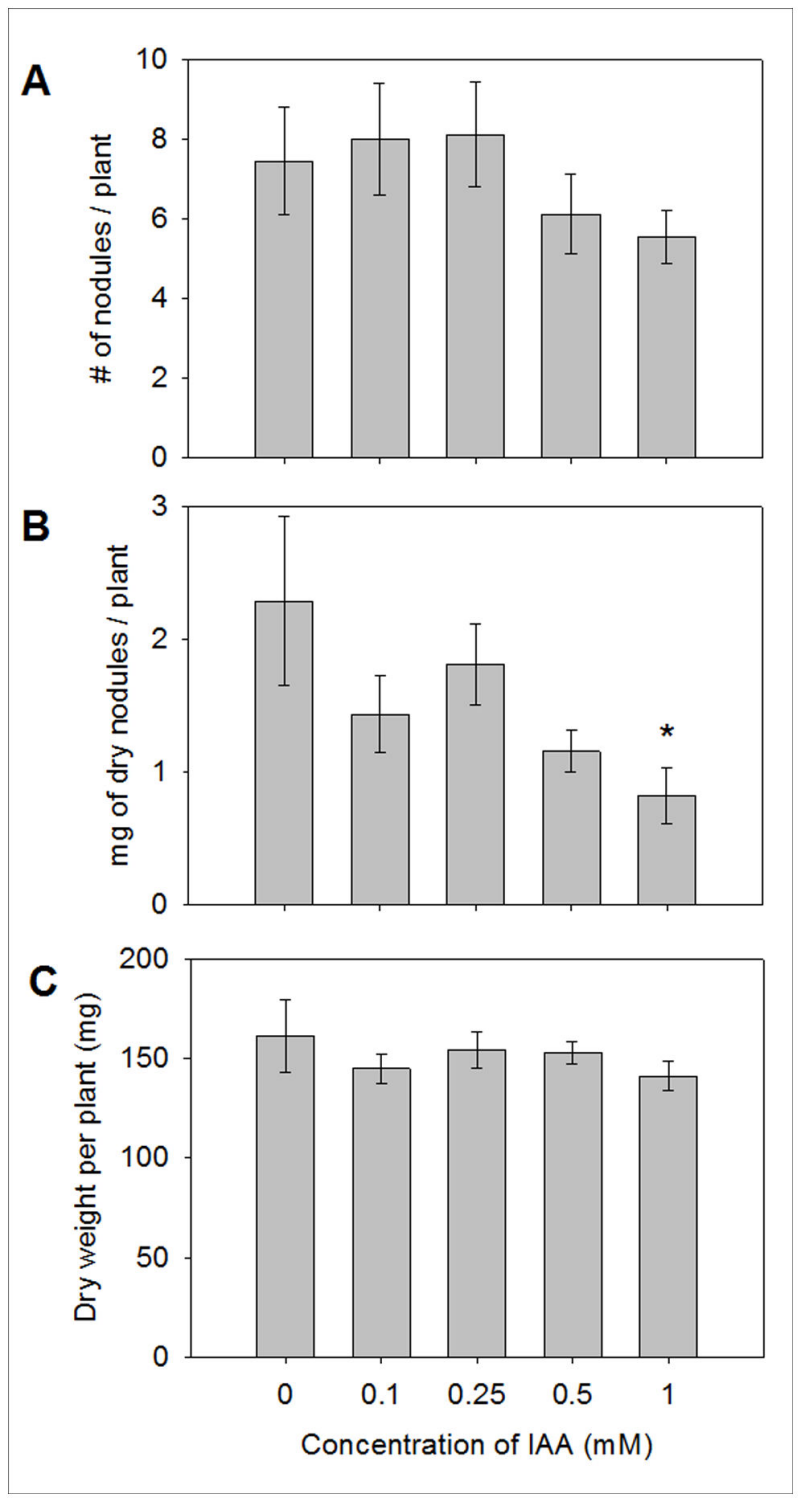
Effect of IAA pretreatment on soybean nodulation by *B. japonicum*. Panel A, B, and C present the number of nodules, nodule dry weight, and plant dry weight, respectively. For measuring dry weights of nodules and plants, samples were dried at 70 °C for 3 days. An asterisk (*) indicates that the value is statistically different (*P* = 0.044) from the control value based on Student’s t-test. Values are the mean ± the standard error of the mean for 9 plants.

We also examined the effect of pretreatment with various concentrations of IAA on the ability of *B. japonicum* to nodulate soybean roots. None of the pretreatments affected the number of nodules, nodule weight, or plant weight (Figure 7), except for the 1 mM IAA condition in the dry nodule weight (Figure 7B): 2.29 ± 0.64 mg of dry nodule weight per plant for the control (no treatment) vs. 0.82 ± 0.21 mg for the 1 mM IAA treatment. The dry weight of nodules slightly decreased when the soybean was inoculated with *B. japonicum* cells pretreated with 1 mM IAA. There was also no delayed nodulation with IAA pretreatments (data not shown). No influence of IAA pretreatments on the whole plant dry weight (Figure 7C) indicates that *B. japonicum* cells pretreated with IAA did not affect the plant growth compared to non-treated cells (Figure 7C).

## Discussion

After evaluating the effects of IAA on the growth and survival parameters of *B. japonicum* across a range of concentrations, we concluded that 1 mM was appropriate for further experimentation due to its intermediate effects on growth parameters (Figure 1). A similar growth study was done to choose an appropriate concentration of IAA for transcriptome profiling of *S. meliloti*, another nitrogen-fixing symbiont [18]. Imperlini et al. chose 0.5 mM IAA after testing the effects of different IAA concentrations (0.05-2.0 mM) on *S. meliloti* growth [18]. Similarly, the same concentration (1 mM IAA) was used for transcriptome profiling of *A. brasilense* [7]. *B. japonicum*, *S.* meliloti, and *A. brasilense* share many physiological and genetic traits, such as soil habitation, nitrogen fixation capability, and IAA metabolism. Apparently the IAA concentrations used in these studies are higher than those have been reported to occur within root nodules (0.58 µg of IAA per g of nodules) [11]. However, local IAA concentrations in the millimolar range have been reported for other legume plant tissues. For example, Figueiredo et al. [44] measured IAA concentrations in the leaves of *Rhizobium*-inoculated common bean *Phaseolus vulgaris* L. of up to 187 µg/ml (ca. 1 mM) of IAA. In addition to plant tissues, levels of IAA experienced by *B. japonicum* in the rhizosphere could be relatively high since 80% of the bacteria isolated from the rhizosphere are IAA producers [45].

IAA exposure affected a rather large portion of the *B. japonicum* genome (15.5%). A generalized stress response would accurately summarize the transcriptional responses of *B. japonicum* to 1 mM IAA. Heat-shock proteins, cold-shock proteins, and molecular chaperones which are associated with maintaining protein integrity within a cell were up-regulated. Similarly, the generalized stress response was also observed in IAA-exposed *E. coli* [15]. In a related study examining the transcriptome analysis of *A. brasilense*, genes associated with ribosomal proteins and the alternative, high-affinity cytochrome *c* (cbb3) oxidase were down-regulated and genes associated with the synthesis of surface and external carbohydrates were up-regulated which is consistent with our transcriptional data [7] (Table S3). In addition, EPS biosynthesis genes and some oxidative stress response genes were also induced (Table 1). EPS production in bacteria has been shown to increase as a result of various types of stresses such as desiccation [25,46], cytotoxic immune responses [47], superoxide mediated-oxidative stress [32] and cold stress [48]. The enhanced EPS production in response to the phytohormone IAA was in good agreement with increased transcription of polysaccharide biosynthesis genes in *B. japonicum*. More interestingly, if the acidic carbohydrate portion is considered alone, a large effect is evident by the 9-fold increase in acidic sugar content. In *Pseudomonas putida* and *Pseudomonas aeruginosa*, the acidic sugar content of the EPS was associated with biofilm formation and EPS integrity, which are essential for bacteria inhabiting the soil in which they are exposed to fluctuating environmental and potentially stressful conditions [49,50]. Uronic acids such as alginate, which is a polymer of D-mannuronic acid and L-guluronic acid, have been linked to tolerance to desiccation and associated oxidative stresses in *P. putida*, a typical soil bacterium [31,46]. Therefore, it will be interesting to reveal the structure of acidic EPSs produced by *B. japonicum* in response to the phytohormone IAA.

Although most of the genes associated with energy metabolism were repressed, the two subunits of an indole pyruvate ferredoxin oxidoreductase protein encoded by bll3410 and bll3411 were highly induced (Table 1). This was also a prominent gene induced in *A. brasilense* in response to 1 mM IAA [7]. This enzyme has been demonstrated to be a major cytoplasmic protein in the anaerobic archaeon *Pyrococcus furiosus* and catalyzes the oxidative decarboxylation of aryl pyruvates [51]. These two loci are candidates for further studies due to their presumable roles in IAA metabolism by *B. japonicum*. Another locus (blr4158) involved in the oxygen-dependent catabolism of IAA which encodes a probable tryptophan 2,3-dioxygenase [13] was moderately induced.

Our transcriptomic data revealed the induction of several heat shock, cold shock, EPS biosynthetic and molecular chaperone proteins as stated before. In order to link gene expression to phenotype, we performed various cell viability assays in response to heat, cold, osmotic, oxidative, and desiccation stresses. We found an increased ability to tolerate these stresses, thereby confirming the inferred promotion of an enhanced stress-tolerant phenotype when exposed to IAA. Our results were in agreement with those from a previous study on *E. coli* in which Bianco et al. found enhanced stress tolerance when the bacteria were pretreated with IAA across a variety of stress conditions [15]. In contrast, the expression of 43 chemotaxis- and flagella-related genes significantly decreased in response to the IAA treatment (Table S3). Our previous functional genomics studies on nutrient limitation and osmotic stress also revealed reduced motility of *B. japonicum* [22]. Possibly, *B. japonicum* cells save energy by shutting off their motility functions, which may implicate an energy conservation strategy to survive a stressful condition by producing more stress-related proteins.

Because the experimental agent is a phytohormone, the expression of genes associated with the bacteroid or symbiotic state could be relevant. A few genes involved in early stages of symbiosis were differentially expressed (Table 1). However, it could be difficult to predict the effects of IAA in this category, since there were relatively equal numbers of up- and down-regulated genes (Table 1). Similarly, some nitrogen fixation-related genes are also up- or down-regulated. In addition to gene expression data, the nodulation assay indicates that IAA pretreatment did not impact nodulation capability and efficiency (Figure 7). We tested common symbiosis parameters such as nodule numbers, nodule dry weight, and plant dry weight, but did not find any significant differences across a range of concentrations except nodule dry weight at 1 mM IAA. Therefore, exogenous IAA may not modulate *B. japonicum*’s ability to invade root cells, but may affect physiological responses to other environmental cues that this bacterium is likely to encounter in the rhizosphere. Nonetheless, it has been observed that IAA is produced by bacteroids and accumulates in soybean nodules [11]. In addition, nodulation abilities of both a mutant *B. japonicum* strain which could synthesize IAA asymbiotically and an engineered *S. meliloti* mutant were shown to be greatly enhanced relative to controls [52,53]. But these were endogenous sources of IAA which demonstrated the ability of a microorganism to manipulate its host plant’s physiology. Exogenous IAA at the given condition in our study appears to have a greater role in making *B. japonicum* more resistant to environmental stresses as opposed to enhanced nodulation. The effect of endogenous IAA production by *B. japonicum* on the soybean plant is under investigated still.

Exposure to IAA could also be likely to affect the outcome of competition in the rhizosphere. Little information on competition, if any, is available. But from our stress tolerance analysis, IAA exposure would theoretically improve *B. japonicum*’s ability to survive and respond to perturbations in the environment. Imperlini et al. also demonstrated effects of IAA on long-term survival of *S. meliloti* [18]. Both the IAA-treated *S. meliloti* wild type and the engineered *S. meliloti* mutant showed increased survival capability compared to the wild type without IAA treatment. Thus, some method of assaying competition among other IAA-producing soil microorganisms such as *S. meliloti*, *A. brasilense*, and *A. tumefaciens* would be interesting to examine whether their co-existence has a synergetic or negative effect.

Both transcriptional and physiological data correlate well with the hypothesis that symbiotic bacteria that can reside in association with a host plant possess compensatory mechanisms for the potential and inevitable perturbation of physiological equilibrium due to the transition from a free-living to an endosymbiont state as well as any threat to cell viability by virtue of its close association with the host plant. Taken together, the transcriptional response is more consistent with a generalized stress response or a preparation for conditions that are inherent to the association with a host plant than it is to promoting nodulation efficiency. Furthermore, a gene expression profile of *B. japonicum* in response to IAA will serve as a reference for further experimental design to study the role of auxins in the context of a symbiotic plant-microbe interaction.

## Supporting Information

Table S1 **Gene specific primers used in the qRT-PCR analysis.** The average efficiency of PCR amplification was greater than 90% for all primer sets used in this study.
(PDF)

Table S2 **Average PCR efficiency for the qRT-PCR analysis.**
(PDF)

Table S3 **Functional classification of genes differentially expressed in response to 1 mM Indole-3-Acetic Acid (IAA).** The cut-off threshold was 2.0-fold with q value less than 5%. Positive values in fold change indicate up-regulation, while negative values indicate down-regulation. Functional levels were adopted from the rhizobase (http://genome.kazusa.or.jp/rhizobase/Bradyrhizobium/genes/category).
(PDF)
