# Supplementary material for: Effects of Indole-3-Acetic Acid on the Transcriptional Activities and Stress Tolerance of Bradyrhizobium japonicum
Source: PLoS One. 2013 Oct 2;8(10):e76559. doi: 10.1371/journal.pone.0076559 (PMC3788728; doi:10.1371/journal.pone.0076559)
Supplement: Table S1 — Gene specific primers used in the qRT-PCR analysis. The average efficiency of PCR amplification was greater than 90% for all primer sets used in this study. (PDF) [file pone.0076559.s001.pdf]

Table S1. Gene specific primers used in the qRT-PCR analysis.

Primer	Sequence (5' → 3')	Gene Description
bll3411(*iorA*)F	TGCCGTGTTCAACAAGCATGAC	indolepyruvate ferredoxin oxidoreductase
bll3411(*iorA*)R	AATTGTTGGTCTTGCGCCGCTT	alpha subunit
bll3410(*iorB*)F	TCTTGCCAACGCCATGACCTAT	indolepyruvate ferredoxin oxidoreductase
bll3410(*iorB*)R	ATGCATGAACTCGGTGGTCTGGA	beta subunit
blr4158F	TCTTCGAGCAGCTCAATAACGCCT	probable tryptophan 2,3-dioxygenase
blr4158R	GCGGTTGCCAAGCAGATATTCGAT	
blr1499(*exoN*)F	AAAATCCGCAAAGCCGTATT	UTP-glucose-1-phosphate uridylyltransferase
blr1499(*exoN*)R	CGAAGATGAAGTGCTCGATG	
bll1186(*atpB'*)F	AGTTCGCTTTCATAGGCCTTCAGC	FoF1 ATP synthase B' chain
bll1186(*atpB'*)R	ATCGAAGCGCGTCAGAACAAGA	
blr2485F	CGCCGTGATTTCTTATTCGT	rieske iron-sulfur protein
blr2485R	GCGCCAGAATACCTTGATGT	
blr1171(*coxA*)F	AACATCTCGTTCTGGCTGCT	cytochrome C oxidase subunit I
blr1171(*coxA*)R	CGTGGTGATGAAGTTGATGG	
bll0631(*parA*)F	TCAACCTTCTGACGGTGAACGC	chromosome partitioning protein A (*parA*)
bll0631(*parA*)R	TGCAGCAATTGCGACAGACCTT	for normalization

The average efficiency of PCR amplification was greater than 90% for all primer sets used in this study.
