# Supplementary material for: Effects of Indole-3-Acetic Acid on the Transcriptional Activities and Stress Tolerance of Bradyrhizobium japonicum
Source: PLoS One. 2013 Oct 2;8(10):e76559. doi: 10.1371/journal.pone.0076559 (PMC3788728; doi:10.1371/journal.pone.0076559)
Supplement: Table S2 — Average PCR efficiency for the qRT-PCR analysis. (PDF) [file pone.0076559.s002.pdf]

Table S2. Average PCR efficiency for the qRT-PCR analysis.

	Average qPCR efficiency (%)	
Gene name	Control	IAA treatment
bll3411 (*iorA*)	97	104
bll3410 (*iorB*)	103	99
blr4158	99	110
blr1499 (*exoN*)	101	110
bll1186 (*atpB'*)	98	97
blr2485	102	90
blr1171 (*coxA*)	109	104
bll0631 (*parA*)	107	103
