# Supplementary material for: Effects of Indole-3-Acetic Acid on the Transcriptional Activities and Stress Tolerance of Bradyrhizobium japonicum
Source: PLoS One. 2013 Oct 2;8(10):e76559. doi: 10.1371/journal.pone.0076559 (PMC3788728; doi:10.1371/journal.pone.0076559)
Supplement: Table S3 — Functional classification of genes differentially expressed in response to 1 mM Indole-3-Acetic Acid (IAA). The cut-off threshold was 2.0-fold with q value less than 5%. Positive values in fold change indicate up-regulation, while negative values indicate down-regulation. Functional levels were adopted from the rhizobase (http://genome.kazusa.or.jp/rhizobase/Bradyrhizobium/genes/category). (PDF) [file pone.0076559.s003.pdf]

Table S3. Functional classification of genes differentially expressed in response to 1 mM Indole-3-Acetic Acid (IAA).
The cut-off threshold was 2.0-fold with q value less than 5%.
Positive values in fold change indicate up-regulation, while negative values indicate down-regulation.
Functional levels were adopted from the rhizobase (http://genome.kazusa.or.jp/rhizobase/Bradyrhizobium/genes/category).

Locus ID	Fold change	q-value(%)	Functional Leve I	Functional Level II	Functional Level III (Description)
blr2068	3.56	0.00	Amino Acid Biosynthesis	Aspartate Family	5-methyltetrahydropteroyltriglutamate-homocysteine S-methyltransferase
blr6687	3.43	0.00	Amino Acid Biosynthesis	Serine Family/Sulfur Assimilation	putative phosphoglycerate dehydrogenase
blr6742	2.79	0.00	Amino Acid Biosynthesis	Glutamate Family/Nitrogen Assimilation	putative glutamate synthase small subunit
blr4158	2.61	0.00	Amino Acid Biosynthesis	Aromatic Amino Acid Family	probable tryptophan 2,3-dioxygenase
bll4682	2.26	0.00	Amino Acid Biosynthesis	Serine Family/Sulfur Assimilation	cysteine synthase
blr2994	2.07	0.00	Amino Acid Biosynthesis	Glutamate Family/Nitrogen Assimilation	ethanolamine ammonia-lyase heavy chain
blr3064	2.06	0.00	Amino Acid Biosynthesis	Branched Chain Family	succinyl-diaminopimelate desuccinylase
blr4687	-4.32	0.00	Amino Acid Biosynthesis	Aspartate Family	aspartate-semialdehyde dehydrogenase
bll6497	-3.74	0.00	Amino Acid Biosynthesis	Branched Chain Family	ketol-acid reductoisomerase
blr4169	-2.98	0.00	Amino Acid Biosynthesis	Glutamate Family/Nitrogen Assimilation	glutamine synthetase II
bll1105	-2.49	0.00	Amino Acid Biosynthesis	Serine Family/Sulfur Assimilation	O-succinylhomoserine sulfhydrylase
blr7454	-2.39	0.00	Amino Acid Biosynthesis	Glutamate Family/Nitrogen Assimilation	delta 1-pyrroline-5-carboxylate reductase
blr4949	-2.37	0.00	Amino Acid Biosynthesis	Glutamate Family/Nitrogen Assimilation	glutamine synthetase I
blr0746	-2.34	0.00	Amino Acid Biosynthesis	Aromatic Amino Acid Family	tryptophan synthase alpha subunit
blr3133	-2.32	0.00	Amino Acid Biosynthesis	Glutamate Family/Nitrogen Assimilation	glutamate-ammonia-ligase adenylyltransferase
blr0745	-2.31	0.00	Amino Acid Biosynthesis	Aromatic Amino Acid Family	tryptophan synthase beta subunit
blr3214	-2.26	0.00	Amino Acid Biosynthesis	Glutamate Family/Nitrogen Assimilation	nitric oxide reductase subunit C
blr7743	-2.26	0.00	Amino Acid Biosynthesis	Glutamate Family/Nitrogen Assimilation	glutamate synthase large subunit
bll6501	-2.24	0.00	Amino Acid Biosynthesis	Branched Chain Family	acetolactate synthase small subunit
blr8104	-2.14	0.00	Amino Acid Biosynthesis	Aspartate Family	2,3,4,5-tetrahydropyridine-2-carboxylate N-succinyltransferase
blr1399	-2.04	0.00	Amino Acid Biosynthesis	Aspartate Family	homoserine O-acetyltransferase
bll0501	-2.02	0.00	Amino Acid Biosynthesis	Aspartate Family	aspartate-semialdehyde dehydrogenase
blr3215	-2.00	0.00	Amino Acid Biosynthesis	Glutamate Family/Nitrogen Assimilation	nitric oxide reductase subunit B
blr8106	-2.00	0.00	Amino Acid Biosynthesis	Branched Chain Family	succinyl-diaminopimelate desuccinylase
bll4149	5.85	0.00	Biosynthesis of cofactors, prosthetic groups, and carriers	Thioredoxin, glutaredoxin, and glutathione	putative glutathione peroxidase
blr2939	3.00	0.00	Biosynthesis of cofactors, prosthetic groups, and carriers	Thioredoxin, glutaredoxin, and glutathione	glutathione S-transferase
blr0583	2.95	0.00	Biosynthesis of cofactors, prosthetic groups, and carriers	Thioredoxin, glutaredoxin, and glutathione	gamma-glutamyltranspeptidase
bll6732	2.71	0.00	Biosynthesis of cofactors, prosthetic groups, and carriers	Thioredoxin, glutaredoxin, and glutathione	probable glutathione peroxidase
bll6273	2.70	0.00	Biosynthesis of cofactors, prosthetic groups, and carriers	Biotin	probable biotin sulfoxide reductase
bll0751	2.58	0.00	Biosynthesis of cofactors, prosthetic groups, and carriers	Thioredoxin, glutaredoxin, and glutathione	thioredoxin C-1
bll3136	2.39	0.00	Biosynthesis of cofactors, prosthetic groups, and carriers	Folic acid	formate dehydrogenase alpha subunit
blr2308	2.39	0.00	Biosynthesis of cofactors, prosthetic groups, and carriers	Molybdopterin	putative molybdopterin biosynthesis protein
bll0668	2.19	0.00	Biosynthesis of cofactors, prosthetic groups, and carriers	Thioredoxin, glutaredoxin, and glutathione	glutathione synthetase
bll0180	2.04	0.00	Biosynthesis of cofactors, prosthetic groups, and carriers	Cobalamin, heme, phycobilin and porphyrin	cobalt insertion protein
bll7086	-5.77	0.00	Biosynthesis of cofactors, prosthetic groups, and carriers	Cobalamin, heme, phycobilin and porphyrin	anaerobic coproporphyrinogen III oxidase
bll5026	-4.33	0.00	Biosynthesis of cofactors, prosthetic groups, and carriers	Carotenoid	H+ translocating pyrophosphate synthase
bll5029	-3.14	0.00	Biosynthesis of cofactors, prosthetic groups, and carriers	Riboflavin	riboflavin synthase beta chain
blr0469	-2.54	0.00	Biosynthesis of cofactors, prosthetic groups, and carriers	Cobalamin, heme, phycobilin and porphyrin	heme exporter protein C
bll2481	-2.40	0.00	Biosynthesis of cofactors, prosthetic groups, and carriers	Cobalamin, heme, phycobilin and porphyrin	coproporphyrinogen III oxidase
bll5031	-2.27	0.00	Biosynthesis of cofactors, prosthetic groups, and carriers	Riboflavin	riboflavin biosynthesis protein
bll2542	-2.25	0.00	Biosynthesis of cofactors, prosthetic groups, and carriers	Quinolinate	quinolinate synthetase A
blr1172	-2.20	0.00	Biosynthesis of cofactors, prosthetic groups, and carriers	Cobalamin, heme, phycobilin and porphyrin	putative heme O synthase
bll1162	-2.10	0.00	Biosynthesis of cofactors, prosthetic groups, and carriers	Thioredoxin, glutaredoxin, and glutathione	glutathione S-transferase
bll5510	3.36	0.00	Cell envelope	Membranes, lipoproteins and porins	outer-membrane immunogenic protein precursor
blr0372	3.25	0.00	Cell envelope	Surface polysaccharides, lipopolysaccharides and antigens	putative succinoglycan biosynthesis protein
blr8158	2.82	0.00	Cell envelope	Murein sacculus and peptidoglycan	murein endopeptidase
bll4321	2.78	0.00	Cell envelope	Membranes, lipoproteins and porins	putative outer membrane channel lipoprotein
bll4743	2.73	0.00	Cell envelope	Membranes, lipoproteins and porins	putative lipoprotein
bll7469	2.24	0.00	Cell envelope	Membranes, lipoproteins and porins	putative outer-membrane immunogenic protein precursor
blr3405	2.21	0.00	Cell envelope	Murein sacculus and peptidoglycan	D-alanine--D-alanine ligase
blr5253	2.20	0.00	Cell envelope	Surface polysaccharides, lipopolysaccharides and antigens	putative polysaccharide deacetylase
blr4700	-18.47	0.00	Cell envelope	Membranes, lipoproteins and porins	putative outer-membrane immunogenic protein precursor
bll6888	-10.76	0.00	Cell envelope	Membranes, lipoproteins and porins	putative porin
blr4701	-7.57	0.00	Cell envelope	Membranes, lipoproteins and porins	putative outer-membrane immunogenic protein precursor
blr1311	-6.33	0.00	Cell envelope	Membranes, lipoproteins and porins	outer membrane protein
blr2272	-4.95	0.00	Cell envelope	Membranes, lipoproteins and porins	putative porin precursor
blr4715	-3.42	0.00	Cell envelope	Membranes, lipoproteins and porins	putative outer-membrane immunogenic protein precursor
bll6025	-3.12	0.00	Cell envelope	Membranes, lipoproteins and porins	probable outer membrane protein B
blr5423	-2.95	0.00	Cell envelope	Surface polysaccharides, lipopolysaccharides and antigens	probable dTDP-glucose-4,6 dehydratase
bll5925	-2.79	0.00	Cell envelope	Surface polysaccharides, lipopolysaccharides and antigens	putative capsular polysaccharide biosynthesis protein
blr7695	-2.78	0.00	Cell envelope	Membranes, lipoproteins and porins	putative quter-membrane immunogenic protein precursor
blr5971	-2.36	0.00	Cell envelope	Surface polysaccharides, lipopolysaccharides and antigens	putative polysaccharide biosynthesis related protein
blr3745	-2.31	0.00	Cell envelope	Murein sacculus and peptidoglycan	periplasmic mannitol-binding protein
bll4853	-2.10	0.00	Cell envelope	Membranes, lipoproteins and porins	putative outer membrane protein
bsl8249	6.02	0.00	Cellular processes	Chaperones	cold shock protein
bll0729	3.92	0.00	Cellular processes	Chaperones	small heat shock protein
bsr0210	3.32	0.00	Cellular processes	Detoxification	glutaredoxin
bll3872	3.27	0.00	Cellular processes	Protein and peptide secretion	HlyD family secretion protein
blr2696	3.18	0.00	Cellular processes	Detoxification	cytochrome C peroxidase
blr5220	3.13	0.00	Cellular processes	Chaperones	small heat shock protein
blr7243	2.85	0.00	Cellular processes	Detoxification	putative cytochrome P450
blr1100	2.81	0.00	Cellular processes	Chaperones	heat shock protein 33
bll4327	2.79	0.00	Cellular processes	Chemotaxis	putative methyl-accepting chemotaxis protein
blr3970	2.73	0.00	Cellular processes	Cell division	cell division protein
blr2450	2.43	0.00	Cellular processes	Chaperones	protease heat shock protein
blr5308	2.41	0.00	Cellular processes	Detoxification	anti-oxidant protein
blr5227	2.39	0.00	Cellular processes	Chaperones	heat shock protein
blr5233	2.38	0.00	Cellular processes	Chaperones	small heat shock protein
bll5184	2.36	0.00	Cellular processes	Detoxification	putative cytochrome P460
bll6987	2.27	0.00	Cellular processes	Protein and peptide secretion	hypothetical signal peptide protein
blr7740	2.21	0.00	Cellular processes	Chaperones	small heat shock protein
blr5226	2.17	0.00	Cellular processes	Chaperones	heat shock protein
blr5221	2.08	0.00	Cellular processes	Chaperones	small heat shock protein
bll6865	-85.68	0.00	Cellular processes	Chemotaxis	flagellin
bll6866	-40.56	0.00	Cellular processes	Chemotaxis	flagellin
bll6876	-35.86	0.00	Cellular processes	Chemotaxis	flagellar basal-body rod protein
bll6858	-32.71	0.00	Cellular processes	Chemotaxis	flagellar hook protein
bll6857	-30.12	0.00	Cellular processes	Chemotaxis	hook associated protein I homolog
bll6856	-29.84	0.00	Cellular processes	Chemotaxis	probable flagellar hook-associated protein
bll6873	-20.33	0.00	Cellular processes	Chemotaxis	flagellar basal-body rod protein
bll6875	-16.35	0.00	Cellular processes	Chemotaxis	flagellar basal-body rod protein
bll6854	-15.87	0.00	Cellular processes	Chemotaxis	flagellin synthesis repressor protein
bsl6853	-14.82	0.00	Cellular processes	Chemotaxis	hook formation protein
bll6862	-14.56	0.00	Cellular processes	Chemotaxis	probable flagellar motor protein
blr6884	-12.03	0.00	Cellular processes	Chemotaxis	flagellar basal body rod protein
bll6878	-10.38	0.00	Cellular processes	Chemotaxis	probable flagellar motor switch protein
blr0576	-10.36	0.00	Cellular processes	Chemotaxis	putative methyl-accepting chemotaxis protein
bll6861	-10.35	0.00	Cellular processes	Chemotaxis	probable chemotaxis protein precursor
bll6877	-10.24	0.00	Cellular processes	Chemotaxis	flagellar biosynthetic protein
bll6855	-10.04	0.00	Cellular processes	Chemotaxis	probable flagellar protein
bll6879	-8.32	0.00	Cellular processes	Chemotaxis	probable flagellar motor switch protein
bll6871	-8.26	0.00	Cellular processes	Chemotaxis	flagellar P-ring protein precursor
bsl6852	-7.97	0.00	Cellular processes	Chemotaxis	flagellar biosynthetic protein

bll6872	-7.08	0.00	Cellular processes	Chemotaxis	probable flagellar protein
bll6874	-6.70	0.00	Cellular processes	Chemotaxis	flagellar hook-basal body complex protein
bll6885	-5.04	0.00	Cellular processes	Chemotaxis	flagellum-specific ATP synthase
bll6869	-4.34	0.00	Cellular processes	Chemotaxis	flagellar L-ring protein precursor
bll6864	-4.23	0.00	Cellular processes	Chemotaxis	flagellar M-ring protein
bsl6587	-4.10	0.00	Cellular processes	Chemotaxis	components of type IV pilus pilin subunit
bsl1445	-4.05	0.00	Cellular processes	Chaperones	cold shock protein
bsl3986	-4.02	0.00	Cellular processes	Chaperones	cold shock protein
bll6850	-3.98	0.00	Cellular processes	Chemotaxis	probable flagellar biosynthetic protein
bll6851	-3.63	0.00	Cellular processes	Chemotaxis	flagellar biosynthesis protein
bll6867	-3.62	0.00	Cellular processes	Chemotaxis	flagellar biosynthetic protein
blr5828	-3.49	0.00	Cellular processes	Chemotaxis	flagellar basal-body rod protein
bll2496	-3.33	0.00	Cellular processes	Cell division	chromosome segregation protein
blr1813	-3.21	0.00	Cellular processes	Protein and peptide secretion	RhcJ protein
blr4635	-3.20	0.00	Cellular processes	Chaperones	chaperonin GroEL
blr2130	-3.18	0.00	Cellular processes	Plasmid transfer	similar to conjugal transfer protein traG
blr5827	-3.13	0.00	Cellular processes	Chemotaxis	flagellar basal-body rod protein
blr6997	-2.77	0.00	Cellular processes	Chemotaxis	probable flagellar hook assembly protein
blr5838	-2.75	0.00	Cellular processes	Chemotaxis	flagellar P-ring protein precursor
bll4735	-2.70	0.00	Cellular processes	Protein and peptide secretion	protein-export membrane protein
blr2349	-2.67	0.00	Cellular processes	Chemotaxis	chemotaxis two-component response regulator
bsl5417	-2.66	0.00	Cellular processes	Protein and peptide secretion	preprotein translocase
bll4736	-2.65	0.00	Cellular processes	Protein and peptide secretion	preprotein tranlocase protein
bll4945	-2.58	0.00	Cellular processes	Protein and peptide secretion	trigger factor
blr2344	-2.52	0.00	Cellular processes	Chemotaxis	probable chemotaxis protein
bll5941	-2.40	0.00	Cellular processes	Cell division	putative partition protein
blr0929	-2.39	0.00	Cellular processes	Chemotaxis	putative methyl accepting chemotaxis protein
blr5830	-2.38	0.00	Cellular processes	Chemotaxis	flagellar L-ring protein precursor
bll4734	-2.37	0.00	Cellular processes	Protein and peptide secretion	protein-export membrane protein
bll6537	-2.36	0.00	Cellular processes	Detoxification	putative cytochrome P450
blr2342	-2.36	0.00	Cellular processes	Chemotaxis	chemotaxis two-component response regulator
blr1819	-2.33	0.00	Cellular processes	Protein and peptide secretion	RhcR protein
blr7533	-2.29	0.00	Cellular processes	Chaperones	60 KDA chaperonin
bll0667	-2.19	0.00	Cellular processes	Chemotaxis	CtpC protein
blr2346	-2.14	0.00	Cellular processes	Chemotaxis	probable chemotaxis protein
bll0664	-2.11	0.00	Cellular processes	Chemotaxis	CtpF protein
bll1470	-2.09	0.00	Cellular processes	Chemotaxis	putative methyl accepting chemotaxis protein
blr6022	-2.07	0.00	Cellular processes	Protein and peptide secretion	probable general secretion pathway protein D
bll6868	-2.07	0.00	Cellular processes	Chemotaxis	flagellar protein
blr2343	-2.06	0.00	Cellular processes	Chemotaxis	chemotaxis two-component sensor histidine kinase
bll7789	-2.06	0.00	Cellular processes	Chaperones	probable chaperonine heat shock hsp90 proteins family
blr2694	9.20	0.00	Central intermediary metabolism	Nitrogen fixation	VirG-like two component response regulator
blr2037	6.85	0.00	Central intermediary metabolism	Nitrogen fixation	nif-specific regulatory protein
bll6928	5.04	0.00	Central intermediary metabolism	Other	HypD' protein
blr8272	4.19	0.00	Central intermediary metabolism	Polysaccharides and glycoproteins	probable phosphoglucomutase phosphomannomutase protein
blr3397	3.64	0.00	Central intermediary metabolism	Nitrogen metabolism	nitrilase
bsl6935	3.52	0.00	Central intermediary metabolism	Other	HupI protein
blr3126	3.11	0.00	Central intermediary metabolism	Nitrogen fixation	cytochrome C-type biogenesis protein
blr1769	3.08	0.00	Central intermediary metabolism	Nitrogen fixation	dinitrogenase reductase protein
blr0211	2.59	0.00	Central intermediary metabolism	Nitrogen metabolism	amidohydrolase
blr1755	2.30	0.00	Central intermediary metabolism	Nitrogen fixation	R. etli iscN homolog
bll1167	2.29	0.00	Central intermediary metabolism	Nitrogen fixation	signal peptidase
blr1774	2.22	0.00	Central intermediary metabolism	Nitrogen fixation	flavoprotein
blr1222	2.20	0.00	Central intermediary metabolism	Phosphorus compounds	phosphonate metabolism protein
blr2038	2.18	0.00	Central intermediary metabolism	Nitrogen fixation	electron transfer flavoprotein beta chain
blr1499	2.16	0.00	Central intermediary metabolism	Nitrogen fixation	UTP-glucose-1-phosphate uridylyltransferase
blr3350	2.08	0.00	Central intermediary metabolism	Other	probable carbon monoxide dehydrogenase large chain
blr7578	2.01	0.00	Central intermediary metabolism	Nitrogen fixation	UDP-glucose 4'-epimerase
bll1137	-20.81	0.00	Central intermediary metabolism	Other	probable carbonic anhydrase
blr7089	-6.02	0.00	Central intermediary metabolism	Nitrogen metabolism	respiratory nitrite reductase
bsr2765	-5.46	0.00	Central intermediary metabolism	Nitrogen fixation	cbb3 oxidase subunit IV
blr2763	-5.24	0.00	Central intermediary metabolism	Nitrogen fixation	cytochrome-c oxidase
blr1459	-5.08	0.00	Central intermediary metabolism	Other	urease accessory protein
blr2764	-5.02	0.00	Central intermediary metabolism	Nitrogen fixation	cytochrome-c oxidase
blr1063	-4.59	0.00	Central intermediary metabolism	Nitrogen fixation	putative autoinducer synthase
blr2768	-3.90	0.00	Central intermediary metabolism	Nitrogen fixation	FixH protein
bll4865	-3.85	0.00	Central intermediary metabolism	Other	putative carbonic anhydrase
bll6940	-3.41	0.00	Central intermediary metabolism	Other	HupC protein
blr2767	-3.08	0.00	Central intermediary metabolism	Nitrogen fixation	iron-sulfur cluster-binding protein
blr2766	-3.03	0.00	Central intermediary metabolism	Nitrogen fixation	cbb3 oxidase subunit III
blr5680	-2.80	0.00	Central intermediary metabolism	Polysaccharides and glycoproteins	ribulose-phosphate 3-epimerase
blr2036	-2.54	0.00	Central intermediary metabolism	Nitrogen fixation	oxidoreductase
bll6944	-2.51	0.00	Central intermediary metabolism	Other	HupU protein
blr7759	-2.08	0.00	Central intermediary metabolism	Other	ornithine decarboxylase
blr3125	-2.01	0.00	Central intermediary metabolism	Nitrogen fixation	cytochrome C-type biogenesis protein
bll4072	10.59	0.00	DNA replication, recombination, and repair		replicative DNA helicase
bll0827	4.05	0.00	DNA replication, recombination, and repair		DNA replication and repair protein
bll7808	3.42	0.00	DNA replication, recombination, and repair		methylated-DNA--protein-cysteine methyltransferase
bll1144	2.56	0.00	DNA replication, recombination, and repair		putative ATP dependent DNA ligase
blr2963	2.27	0.00	DNA replication, recombination, and repair		alkylated DNA repair protein
blr0915	2.13	0.00	DNA replication, recombination, and repair		methylated-DNA-[protein]-cysteine S-methyltransferase
bll0830	-4.16	0.00	DNA replication, recombination, and repair		chromosomal replication initiator protein
bll8008	-3.14	0.00	DNA replication, recombination, and repair		similar to recombinase
bll5755	-3.01	0.00	DNA replication, recombination, and repair		RecA protein
blr4377	-2.67	0.00	DNA replication, recombination, and repair		single-stranded-DNA-specific exonuclease
blr5493	-2.23	0.00	DNA replication, recombination, and repair		ATP-dependent DNA helicase
blr6561	-2.21	0.00	DNA replication, recombination, and repair		DNA helicase II
bll5057	-2.20	0.00	DNA replication, recombination, and repair		topoisomerase II
bll4667	-2.13	0.00	DNA replication, recombination, and repair		probable transcription-repair coupling factor
bll4698	-2.07	0.00	DNA replication, recombination, and repair		single-strand DNA binding protein
blr1536	-2.04	0.00	DNA replication, recombination, and repair		holliday junction DNA helicase
blr7041	-2.02	0.00	DNA replication, recombination, and repair		exodeoxyribosylnuclease
bll3411	30.67	0.00	Energy metabolism	Respiration	indolepyruvate ferredoxin oxidoreductase alpha subunit
bll6076	9.53	0.00	Energy metabolism	Pyruvate and acetyl-CoA metabolism	putative acetyl-CoA synthetase
bll3410	7.64	0.00	Energy metabolism	Respiration	indolepyruvate ferredoxin oxidoreductase beta subunit
blr6219	7.53	0.00	Energy metabolism	Pyruvate and acetyl-CoA metabolism	putative aldehyde dehydrogenase
blr0807	3.90	0.00	Energy metabolism	Amino acids and amines	succinate-semialdehyde dehydrogenase
blr6758	3.08	0.00	Energy metabolism	Sugars	probable transaldolase
blr1248	3.06	0.00	Energy metabolism	Respiration	putative thioredoxin reductase
bll4950	3.00	0.00	Energy metabolism	Amino acids and amines	probable L-asparaginase
bll2736	2.97	0.00	Energy metabolism	Pyruvate and acetyl-CoA metabolism	putative aldehyde dehydrogenase protein
bll1530	2.79	0.00	Energy metabolism	Pyruvate and acetyl-CoA metabolism	putative aldehyde dehydrogenase protein
blr6759	2.67	0.00	Energy metabolism	Pentose phosphate pathway	6-phosphogluconate dehydrogenase
blr2816	2.51	0.00	Energy metabolism	Pyruvate and acetyl-CoA metabolism	aldehyde dehydrogenase
bll6137	2.50	0.00	Energy metabolism	Other	quinone oxidoreductase
blr3179	2.50	0.00	Energy metabolism	Amino acids and amines	probable alanine dehydrogenase oxidoreductase protein
bll7906	2.35	0.00	Energy metabolism	Respiration	putative ferredoxin
blr3957	2.32	0.00	Energy metabolism	Amino acids and amines	3-hydroxyisobutyrate dehydrogenase
bll7157	2.19	0.00	Energy metabolism	Sugars	putative glucose 1-dehydrogenase

bll8129	2.07	0.00	Energy metabolism	Sugars	UDP-glucose 6-dehydrogenase
blr4422	2.03	0.00	Energy metabolism	Amino acids and amines	hydroxymethylglutaryl-CoA lyase
blr2316	2.00	0.00	Energy metabolism	Respiration	probable NADH-ubiquinone oxidoreductase chain F
bll1186	-9.46	0.00	Energy metabolism	Respiration	FoF1 ATP synthase B' chain
bll4784	-9.13	0.00	Energy metabolism	Pyruvate and acetyl-CoA metabolism	aldehyde dehydrogenase
bsl1187	-8.59	0.00	Energy metabolism	Respiration	FoF1 ATP synthase C chain
bll1185	-7.52	0.00	Energy metabolism	Respiration	FoF1 ATP synthase B chain
blr1309	-5.62	0.00	Energy metabolism	Pyruvate and acetyl-CoA metabolism	acetyl-coenzyme A synthetase
bll1188	-4.64	0.00	Energy metabolism	Respiration	FoF1 ATP synthase A chain
blr2485	-4.55	0.00	Energy metabolism	Respiration	rieske iron-sulfur protein
blr2486	-3.89	0.00	Energy metabolism	Respiration	cytochrome b/c1 precursor
bsl1189	-3.86	0.00	Energy metabolism	Respiration	FoF1 ATP synthase subunit I
blr3206	-3.65	0.00	Energy metabolism	Sugars	probable aldose 1-epimerase precursor
blr7448	-3.54	0.00	Energy metabolism	Pentose phosphate pathway	ribose-phosphate pyrophosphokinase
bll2210	-3.25	0.00	Energy metabolism	Respiration	multicopper oxidase
bll0441	-3.09	0.00	Energy metabolism	Respiration	ATP synthase gamma chain
bll0439	-3.02	0.00	Energy metabolism	Respiration	ATP synthase epsilon chain
bll0442	-2.88	0.00	Energy metabolism	Respiration	ATP synthase alpha chain
bll3998	-2.84	0.01	Energy metabolism	Amino acids and amines	probable succinate-semialdehyde dehydrogenase [NADP+]
blr4657	-2.82	0.00	Energy metabolism	Sugars	beta-glucosidase
blr4955	-2.55	0.00	Energy metabolism	Respiration	putative cytochrome B561
blr1171	-2.54	0.00	Energy metabolism	Respiration	cytochrome C oxidase subunit I
blr1423	-2.39	0.00	Energy metabolism	Respiration	cytochrome c
blr6128	-2.39	0.00	Energy metabolism	Respiration	cytochrome c552
blr1175	-2.30	0.00	Energy metabolism	Respiration	cytochrome C oxidase subunit III
blr7525	-2.29	0.00	Energy metabolism	Respiration	ATP phosphoribosyltransferase
bll0440	-2.25	0.00	Energy metabolism	Respiration	ATP synthase beta chain
blr6062	-2.25	0.00	Energy metabolism	Respiration	putative cytochrome C6 precursor
blr0573	-2.23	0.00	Energy metabolism	Pyruvate and acetyl-CoA metabolism	acetyl-CoA synthetase
bll2388	-2.22	0.00	Energy metabolism	Respiration	cytochrome c2
bll7126	-2.18	0.00	Energy metabolism	Respiration	NAD(P)+ transhydrogenase
bll7125	-2.16	0.00	Energy metabolism	TCA cycle	NAD(P) transhydrogenase subunit alpha part 2
blr7544	-2.13	0.00	Energy metabolism	Respiration	cytochrome c550
blr1170	-2.05	0.00	Energy metabolism	Respiration	cytochrome C oxidase subunit II
bll7124	-2.05	0.00	Energy metabolism	Respiration	NAD(P)+ transhydrogenase beta chain
blr1656	-2.04	0.00	Energy metabolism	Sugars	putative glycosyl hydrolase
bll4754	-2.00	0.00	Energy metabolism	Sugars	glycosyl hydrolase
blr3402	11.58	0.00	Fatty acid, phospholipid and sterol metabolism		putative crotonobetaine/carnitine-CoA ligase
bll6081	9.76	0.00	Fatty acid, phospholipid and sterol metabolism		putative enoyl-CoA hydratase
blr3414	6.98	0.00	Fatty acid, phospholipid and sterol metabolism		putative 4-hydroxybenzoyl CoA thioesterase
blr3940	6.50	0.00	Fatty acid, phospholipid and sterol metabolism		putative propionyl-CoA carboxylase beta chain
blr6085	5.24	0.00	Fatty acid, phospholipid and sterol metabolism		putative acid--CoA ligase
blr3433	4.02	0.00	Fatty acid, phospholipid and sterol metabolism		putative crotonobetaine/carnitine-CoA ligase
bll7802	4.00	0.00	Fatty acid, phospholipid and sterol metabolism		long-chain-fatty-acid-CoA ligase
blr6087	3.85	0.00	Fatty acid, phospholipid and sterol metabolism		putative 3-hydroxybutyryl-CoA dehydrogenase
bll0116	2.73	0.00	Fatty acid, phospholipid and sterol metabolism		putative enoyl-CoA hydratase
blr3437	2.66	0.00	Fatty acid, phospholipid and sterol metabolism		putative acyl-CoA dehydrogenase
blr5602	2.65	0.00	Fatty acid, phospholipid and sterol metabolism		hippurate hydrolase
bll2783	2.37	0.00	Fatty acid, phospholipid and sterol metabolism		probable enoyl-CoA hydratase
bll7763	2.10	0.00	Fatty acid, phospholipid and sterol metabolism		probable acetoacetate decarboxylase
blr6442	2.04	0.00	Fatty acid, phospholipid and sterol metabolism		probable D-amino acid oxidase
blr5969	-3.15	0.00	Fatty acid, phospholipid and sterol metabolism		putative anaerobic phenylacetate CoA ligase
blr6836	-3.09	0.00	Fatty acid, phospholipid and sterol metabolism		mannitol dehydrogenase
bll6830	-2.58	0.00	Fatty acid, phospholipid and sterol metabolism		mannonate dehydratase
blr4082	-2.48	0.00	Fatty acid, phospholipid and sterol metabolism		malonyl-CoA:acyl carrier protein transacylase
bll5021	-2.45	0.00	Fatty acid, phospholipid and sterol metabolism		fatty acid/phospholipid synthesis protein
bll1200	-2.42	0.00	Fatty acid, phospholipid and sterol metabolism		5-aminolevulinic acid synthase
blr4085	-2.23	0.00	Fatty acid, phospholipid and sterol metabolism		3-oxoacyl-(acyl carrier protein) synthase II
bll1082	-2.16	0.00	Fatty acid, phospholipid and sterol metabolism		2-deoxy-D-gluconate 3-dehydrogenase
bll2619	-2.09	0.00	Fatty acid, phospholipid and sterol metabolism		medium-chain-fatty-acid--CoA ligase
bll7400	-2.04	0.00	Fatty acid, phospholipid and sterol metabolism		acetyl-CoA acetyltransferase
blr2247	-2.03	0.00	Fatty acid, phospholipid and sterol metabolism		probable fatty acid desaturase
bsl7903	56.52	0.00	Hypothetical	Conserved hypothetical protein	hypothetical protein bsl7903
blr4417	52.91	0.00	Hypothetical	No similarity	hypothetical protein blr4417
blr3415	38.04	0.00	Hypothetical	Conserved hypothetical protein	hypothetical protein blr3415
blr0305	27.07	0.00	Hypothetical	No similarity	hypothetical protein blr0305
blr6167	26.33	0.00	Hypothetical	No similarity	hypothetical protein blr6167
bll7902	24.73	0.00	Hypothetical	Conserved hypothetical protein	hypothetical protein bll7902
blr3427	23.31	0.00	Hypothetical	No similarity	hypothetical protein blr3427
bll5555	21.42	0.00	Hypothetical	Conserved hypothetical protein	hypothetical protein bll5555
blr3413	21.42	0.00	Hypothetical	Conserved hypothetical protein	hypothetical protein blr3413
bll6449	19.44	0.00	Hypothetical	Conserved hypothetical protein	hypothetical protein bll6449
bll4983	19.32	0.00	Hypothetical	Conserved hypothetical protein	hypothetical protein bll4983
bll5579	18.12	0.00	Hypothetical	Conserved hypothetical protein	hypothetical protein bll5579
bll6552	16.62	0.00	Hypothetical	Conserved hypothetical protein	hypothetical protein bll6552
bll7487	13.88	0.00	Hypothetical	No similarity	hypothetical protein bll7487
bsl4437	13.04	0.00	Hypothetical	No similarity	hypothetical protein bsl4437
bsl4014	12.42	0.00	Hypothetical	No similarity	hypothetical protein bsl4014
bsr0862	10.55	0.00	Hypothetical	No similarity	hypothetical protein bsr0862
blr0306	10.37	0.00	Hypothetical	Conserved hypothetical protein	hypothetical protein blr0306
bsr4726	10.29	0.00	Hypothetical	No similarity	hypothetical protein bsr4726
bsr6217	9.59	0.00	Hypothetical	No similarity	hypothetical protein bsr6217
bsr3435	9.50	0.00	Hypothetical	No similarity	hypothetical protein bsr3435
bll5835	9.04	0.00	Hypothetical	Conserved hypothetical protein	hypothetical protein bll5835
bll7562	8.90	0.00	Hypothetical	Conserved hypothetical protein	hypothetical protein bll7562
blr6533	8.85	0.00	Hypothetical	Conserved hypothetical protein	hypothetical protein blr6533
bsr6700	8.79	0.00	Hypothetical	No similarity	hypothetical protein bsr6700
bll4882	8.37	0.00	Hypothetical	No similarity	hypothetical protein bll4882
bll2743	8.30	0.00	Hypothetical	Conserved hypothetical protein	hypothetical protein bll2743
bll0836	8.27	0.00	Hypothetical	No similarity	hypothetical protein bll0836
blr1206	8.06	0.00	Hypothetical	No similarity	hypothetical protein blr1206
blr3429	7.89	0.00	Hypothetical	Conserved hypothetical protein	hypothetical protein blr3429
bsr7633	7.54	0.00	Hypothetical	No similarity	hypothetical protein bsr7633
bll4880	7.22	0.00	Hypothetical	Conserved hypothetical protein	hypothetical protein bll4880
bll4985	7.14	0.00	Hypothetical	Conserved hypothetical protein	hypothetical protein bll4985
blr0094	6.69	0.00	Hypothetical	Conserved hypothetical protein	hypothetical protein blr0094
bll4881	6.68	0.00	Hypothetical	Conserved hypothetical protein	hypothetical protein bll4881
blr1387	6.62	0.00	Hypothetical	Conserved hypothetical protein	hypothetical protein blr1387
bll1207	6.55	0.00	Hypothetical	Conserved hypothetical protein	hypothetical protein bll1207
bll7547	6.41	0.00	Hypothetical	Conserved hypothetical protein	hypothetical protein bll7547
bsl0296.2n	6.38	0.00	Hypothetical		hypothetical protein bsl0296.2n
blr5716	6.34	0.00	Hypothetical	Conserved hypothetical protein	hypothetical protein blr5716
blr2935	6.16	0.00	Hypothetical	Conserved hypothetical protein	hypothetical protein blr2935
blr2962	6.03	0.00	Hypothetical	Conserved hypothetical protein	hypothetical protein blr2962
bsl7109	5.93	0.00	Hypothetical	Conserved hypothetical protein	hypothetical protein bsl7109
blr4982	5.83	0.00	Hypothetical	Conserved hypothetical protein	hypothetical protein blr4982
bsr4471	5.77	0.00	Hypothetical	No similarity	hypothetical protein bsr4471
blr0276	5.74	0.00	Hypothetical	Conserved hypothetical protein	hypothetical protein blr0276
bsl6734	5.66	0.00	Hypothetical	Conserved hypothetical protein	hypothetical protein bsl6734

bll5492	5.37	0.00	Hypothetical	Conserved hypothetical protein	hypothetical protein bll5492
bll0233	5.35	0.00	Hypothetical	Conserved hypothetical protein	hypothetical protein bll0233
blr5636	5.17	0.00	Hypothetical	No similarity	hypothetical protein blr5636
bll2700	5.05	0.00	Hypothetical	Conserved hypothetical protein	hypothetical protein bll2700
bll1322.1n	4.92	0.00	Hypothetical		hypothetical protein bll1322.1n
blr5127	4.86	0.00	Hypothetical	No similarity	hypothetical protein blr5127
blr2425	4.77	0.00	Hypothetical	Conserved hypothetical protein	hypothetical protein blr2425
bll5237	4.70	0.00	Hypothetical	No similarity	hypothetical protein bll5237
bll1594	4.68	0.00	Hypothetical	No similarity	hypothetical protein bll1594
blr7935	4.56	0.00	Hypothetical	Conserved hypothetical protein	hypothetical protein blr7935
bll2330	4.54	0.00	Hypothetical	Conserved hypothetical protein	hypothetical protein bll2330
bsl2206	4.50	0.00	Hypothetical	Conserved hypothetical protein	hypothetical protein bsl2206
bll6811	4.50	0.00	Hypothetical	Conserved hypothetical protein	hypothetical protein bll6811
bll1285	4.45	0.00	Hypothetical	No similarity	hypothetical protein bll1285
blr5768	4.42	0.00	Hypothetical	No similarity	hypothetical protein blr5768
bll6754	4.41	0.00	Hypothetical	Conserved hypothetical protein	hypothetical protein bll6754
blr2360	4.39	0.00	Hypothetical	Conserved hypothetical protein	hypothetical protein blr2360
bsr0431	4.39	0.00	Hypothetical	Conserved hypothetical protein	hypothetical protein bsr0431
bll5640	4.36	0.00	Hypothetical	No similarity	hypothetical protein bll5640
bll5372	4.35	0.00	Hypothetical	Conserved hypothetical protein	hypothetical protein bll5372
blr0401	4.29	0.00	Hypothetical	Conserved hypothetical protein	hypothetical protein blr0401
blr8065	4.23	0.00	Hypothetical	Conserved hypothetical protein	hypothetical protein blr8065
bll5323	4.21	0.00	Hypothetical	No similarity	hypothetical protein bll5323
bll7908	4.20	0.00	Hypothetical	Conserved hypothetical protein	hypothetical protein bll7908
blr1229	4.17	0.00	Hypothetical	Conserved hypothetical protein	hypothetical protein blr1229
blr4723	4.13	0.00	Hypothetical	Conserved hypothetical protein	hypothetical protein blr4723
blr4176	4.11	0.00	Hypothetical	No similarity	hypothetical protein blr4176
bll3592	4.11	0.00	Hypothetical	Conserved hypothetical protein	hypothetical protein bll3592
blr4684	4.11	0.00	Hypothetical	Conserved hypothetical protein	hypothetical protein blr4684
blr6086	4.08	0.00	Hypothetical	No similarity	hypothetical protein blr6086
blr6531	4.05	0.00	Hypothetical	Conserved hypothetical protein	hypothetical protein blr6531
blr1402	4.05	0.00	Hypothetical	Conserved hypothetical protein	hypothetical protein blr1402
bll5315.1n	3.99	0.00	Hypothetical		hypothetical protein bll5315.1n
blr5713	3.98	0.00	Hypothetical	Conserved hypothetical protein	hypothetical protein blr5713
bll2462	3.97	0.00	Hypothetical	Conserved hypothetical protein	hypothetical protein bll2462
blr4264.1n	3.87	0.00	Hypothetical		hypothetical protein blr4264.1n
blr6699	3.85	0.00	Hypothetical	No similarity	hypothetical protein blr6699
blr4356	3.83	0.00	Hypothetical	Conserved hypothetical protein	hypothetical protein blr4356
bsl0879	3.78	0.00	Hypothetical	Conserved hypothetical protein	hypothetical protein bsl0879
bsr4559	3.73	0.00	Hypothetical	Conserved hypothetical protein	hypothetical protein bsr4559
blr4686	3.70	0.00	Hypothetical	Conserved hypothetical protein	hypothetical protein blr4686
bll3037	3.70	0.00	Hypothetical	Conserved hypothetical protein	hypothetical protein bll3037
blr2641	3.65	0.00	Hypothetical	Conserved hypothetical protein	hypothetical protein blr2641
bsl0231	3.63	0.00	Hypothetical	No similarity	hypothetical protein bsl0231
bll7635	3.62	0.00	Hypothetical	No similarity	hypothetical protein bll7635
bll5899	3.61	0.00	Hypothetical	Conserved hypothetical protein	hypothetical protein bll5899
bsr8045	3.57	0.00	Hypothetical	Conserved hypothetical protein	hypothetical protein bsr8045
blr7694	3.55	0.00	Hypothetical	Conserved hypothetical protein	hypothetical protein blr7694
blr3860	3.54	0.00	Hypothetical	No similarity	hypothetical protein blr3860
bll7057	3.53	0.00	Hypothetical	Conserved hypothetical protein	hypothetical protein bll7057
blr5292	3.52	0.00	Hypothetical	No similarity	hypothetical protein blr5292
bll6809	3.51	0.00	Hypothetical	Conserved hypothetical protein	hypothetical protein bll6809
blr2592	3.48	0.00	Hypothetical	No similarity	hypothetical protein blr2592
blr1492	3.41	0.00	Hypothetical	Conserved hypothetical protein	hypothetical protein blr1492
bll4879	3.37	0.00	Hypothetical	Conserved hypothetical protein	hypothetical protein bll4879
bll4278	3.35	0.00	Hypothetical	No similarity	hypothetical protein bll4278
blr4433	3.32	0.00	Hypothetical	No similarity	hypothetical protein blr4433
blr0274	3.28	0.00	Hypothetical	No similarity	hypothetical protein blr0274
blr8159	3.27	0.00	Hypothetical	Conserved hypothetical protein	hypothetical protein blr8159
blr4162	3.26	0.00	Hypothetical	Conserved hypothetical protein	hypothetical protein blr4162
bll3144	3.25	0.00	Hypothetical	Conserved hypothetical protein	hypothetical protein bll3144
bll4218	3.25	0.00	Hypothetical	Conserved hypothetical protein	hypothetical protein bll4218
bll4360.1n	3.24	0.00	Hypothetical		hypothetical protein bll4360.1n
bsl2782	3.22	0.00	Hypothetical	Conserved hypothetical protein	hypothetical protein bsl2782
bsr4236	3.19	0.00	Hypothetical	No similarity	hypothetical protein bsr4236
bll7670	3.19	0.00	Hypothetical	No similarity	hypothetical protein bll7670
bll1491	3.19	0.00	Hypothetical	Conserved hypothetical protein	hypothetical protein bll1491
blr2069	3.18	0.00	Hypothetical	No similarity	hypothetical protein blr2069
blr3431	3.15	0.00	Hypothetical	Conserved hypothetical protein	hypothetical protein blr3431
blr4219	3.15	0.00	Hypothetical	Conserved hypothetical protein	hypothetical protein blr4219
bll7529	3.12	0.00	Hypothetical	Conserved hypothetical protein	hypothetical protein bll7529
bll6468	3.08	0.00	Hypothetical	Conserved hypothetical protein	hypothetical protein bll6468
bll7241	3.08	0.00	Hypothetical	No similarity	hypothetical protein bll7241
bll5708	3.08	0.00	Hypothetical	Conserved hypothetical protein	hypothetical protein bll5708
blr5712	3.05	0.00	Hypothetical	Conserved hypothetical protein	hypothetical protein blr5712
bll4089	3.05	0.00	Hypothetical	No similarity	hypothetical protein bll4089
bll6756	3.03	0.00	Hypothetical	Conserved hypothetical protein	hypothetical protein bll6756
blr1284	3.02	0.00	Hypothetical	Conserved hypothetical protein	hypothetical protein blr1284
blr6228	3.00	0.00	Hypothetical	No similarity	hypothetical protein blr6228
bll1897	2.99	0.00	Hypothetical	No similarity	hypothetical protein bll1897
blr4621	2.98	0.00	Hypothetical	No similarity	hypothetical protein blr4621
bll2734	2.98	0.00	Hypothetical	Conserved hypothetical protein	hypothetical protein bll2734
blr5128	2.96	0.00	Hypothetical	Conserved hypothetical protein	hypothetical protein blr5128
bll4725	2.96	0.00	Hypothetical	No similarity	hypothetical protein bll4725
bll4328	2.95	0.00	Hypothetical	Conserved hypothetical protein	hypothetical protein bll4328
blr5637	2.93	0.00	Hypothetical	No similarity	hypothetical protein blr5637
bll1342	2.91	0.00	Hypothetical	Conserved hypothetical protein	hypothetical protein bll1342
blr1245	2.90	0.00	Hypothetical	No similarity	hypothetical protein blr1245
bsr4721	2.89	0.00	Hypothetical	Conserved hypothetical protein	hypothetical protein bsr4721
bsl6617	2.89	0.00	Hypothetical	No similarity	hypothetical protein bsl6617
bll6075	2.87	0.00	Hypothetical	Conserved hypothetical protein	hypothetical protein bll6075
blr2811	2.87	0.00	Hypothetical	Conserved hypothetical protein	hypothetical protein blr2811
blr3407	2.86	0.00	Hypothetical	No similarity	hypothetical protein blr3407
bll7080	2.85	0.00	Hypothetical	No similarity	hypothetical protein bll7080
blr2318	2.84	0.00	Hypothetical	Conserved hypothetical protein	hypothetical protein blr2318
bll1464	2.84	0.00	Hypothetical	Conserved hypothetical protein	hypothetical protein bll1464
blr4759	2.83	0.00	Hypothetical	Conserved hypothetical protein	hypothetical protein blr4759
bsr4434	2.83	0.00	Hypothetical	No similarity	hypothetical protein bsr4434
blr2430.2n	2.82	0.00	Hypothetical		hypothetical protein blr2430.2n
blr2857	2.81	0.00	Hypothetical	Conserved hypothetical protein	hypothetical protein blr2857
blr7528	2.81	0.00	Hypothetical	Conserved hypothetical protein	hypothetical protein blr7528
blr4157	2.78	0.00	Hypothetical	Conserved hypothetical protein	hypothetical protein blr4157
blr4724	2.76	0.00	Hypothetical	Conserved hypothetical protein	hypothetical protein blr4724
bll6615	2.76	0.00	Hypothetical	Conserved hypothetical protein	hypothetical protein bll6615
blr7167.1n	2.76	0.00	Hypothetical		hypothetical protein blr7167.1n
bll4295	2.75	0.00	Hypothetical	Conserved hypothetical protein	hypothetical protein bll4295
blr0056	2.74	0.00	Hypothetical	No similarity	hypothetical protein blr0056
blr3398	2.73	0.00	Hypothetical	Conserved hypothetical protein	hypothetical protein blr3398
blr4382	2.73	0.00	Hypothetical	Conserved hypothetical protein	hypothetical protein blr4382

blr1266	2.73	0.00	Hypothetical	Conserved hypothetical protein	hypothetical protein blr1266
bsl0728	2.73	0.00	Hypothetical	Conserved hypothetical protein	hypothetical protein bsl0728
bll8202	2.72	0.00	Hypothetical	No similarity	hypothetical protein bll8202
blr0866	2.71	0.00	Hypothetical	No similarity	hypothetical protein blr0866
blr5847	2.70	0.00	Hypothetical	Conserved hypothetical protein	hypothetical protein blr5847
blr3761	2.70	0.00	Hypothetical	Conserved hypothetical protein	hypothetical protein blr3761
bll6198	2.70	0.00	Hypothetical	Conserved hypothetical protein	hypothetical protein bll6198
blr6582	2.69	0.00	Hypothetical	Conserved hypothetical protein	hypothetical protein blr6582
bll2443	2.68	0.00	Hypothetical	No similarity	hypothetical protein bll2443
blr6173	2.67	0.00	Hypothetical	No similarity	hypothetical protein blr6173
blr0867	2.65	0.00	Hypothetical	No similarity	hypothetical protein blr0867
blr1267	2.65	0.00	Hypothetical	No similarity	hypothetical protein blr1267
blr2860	2.65	0.00	Hypothetical	Conserved hypothetical protein	hypothetical protein blr2860
bll2446	2.65	0.00	Hypothetical	Conserved hypothetical protein	hypothetical protein bll2446
bll7264	2.64	0.00	Hypothetical	Conserved hypothetical protein	hypothetical protein bll7264
bsl2064	2.61	0.00	Hypothetical	No similarity	hypothetical protein bsl2064
bll3359	2.60	0.00	Hypothetical	Conserved hypothetical protein	hypothetical protein bll3359
blr1408	2.59	0.00	Hypothetical	Conserved hypothetical protein	hypothetical protein blr1408
bll7411	2.59	0.00	Hypothetical	Conserved hypothetical protein	hypothetical protein bll7411
blr6894	2.57	0.00	Hypothetical	No similarity	hypothetical protein blr6894
blr5228	2.55	0.00	Hypothetical	No similarity	hypothetical protein blr5228
blr1283	2.55	0.00	Hypothetical	Conserved hypothetical protein	hypothetical protein blr1283
bll1299	2.55	0.00	Hypothetical	Conserved hypothetical protein	hypothetical protein bll1299
bsl2575	2.55	0.00	Hypothetical	Conserved hypothetical protein	hypothetical protein bsl2575
bll6088	2.55	0.00	Hypothetical	No similarity	hypothetical protein bll6088
bsl7120	2.51	0.00	Hypothetical	Conserved hypothetical protein	hypothetical protein bsl7120
bll5166	2.51	0.00	Hypothetical	Conserved hypothetical protein	hypothetical protein bll5166
blr3696	2.50	0.00	Hypothetical	Conserved hypothetical protein	hypothetical protein blr3696
blr2359	2.49	0.00	Hypothetical	Conserved hypothetical protein	hypothetical protein blr2359
bsl2020	2.49	0.00	Hypothetical	Conserved hypothetical protein	hypothetical protein bsl2020
bsl4623	2.49	0.00	Hypothetical	No similarity	hypothetical protein bsl4623
bll5764	2.48	0.00	Hypothetical	Conserved hypothetical protein	hypothetical protein bll5764
blr5601	2.48	0.00	Hypothetical	Conserved hypothetical protein	hypothetical protein blr5601
blr6907	2.46	0.00	Hypothetical	No similarity	hypothetical protein blr6907
blr6079	2.45	0.00	Hypothetical	Conserved hypothetical protein	hypothetical protein blr6079
blr1265	2.44	0.00	Hypothetical	No similarity	hypothetical protein blr1265
bll1367	2.43	0.00	Hypothetical	Conserved hypothetical protein	hypothetical protein bll1367
bll4833	2.43	0.00	Hypothetical	No similarity	hypothetical protein bll4833
blr2995	2.42	0.00	Hypothetical	Conserved hypothetical protein	hypothetical protein blr2995
bll5875	2.42	0.00	Hypothetical	No similarity	hypothetical protein bll5875
blr1282	2.42	0.00	Hypothetical	Conserved hypothetical protein	hypothetical protein blr1282
bll0813	2.42	0.00	Hypothetical	Conserved hypothetical protein	hypothetical protein bll0813
blr6197	2.41	0.00	Hypothetical	Conserved hypothetical protein	hypothetical protein blr6197
bll1366	2.41	0.00	Hypothetical	No similarity	hypothetical protein bll1366
bsr1749	2.40	0.00	Hypothetical	Conserved hypothetical protein	hypothetical protein bsr1749
blr1340	2.40	0.00	Hypothetical	Conserved hypothetical protein	hypothetical protein blr1340
bll4360.2n	2.40	0.00	Hypothetical		hypothetical protein bll4360.2n
blr7131	2.39	0.00	Hypothetical	Conserved hypothetical protein	hypothetical protein blr7131
bsl1208	2.38	0.00	Hypothetical	Conserved hypothetical protein	hypothetical protein bsl1208
blr7950	2.38	0.00	Hypothetical	Conserved hypothetical protein	hypothetical protein blr7950
blr5512	2.38	0.00	Hypothetical	No similarity	hypothetical protein blr5512
bll7960	2.38	0.00	Hypothetical	Conserved hypothetical protein	hypothetical protein bll7960
blr0058	2.37	0.00	Hypothetical	No similarity	hypothetical protein blr0058
bll8036	2.36	0.00	Hypothetical	No similarity	hypothetical protein bll8036
bsr7707	2.36	0.00	Hypothetical	No similarity	hypothetical protein bsr7707
bll4712	2.36	0.00	Hypothetical	No similarity	hypothetical protein bll472
blr1300	2.36	0.00	Hypothetical	Conserved hypothetical protein	hypothetical protein blr1300
blr2057	2.36	0.00	Hypothetical	No similarity	hypothetical protein blr2057
blr4245	2.35	0.00	Hypothetical	No similarity	hypothetical protein blr4245
bll6093	2.35	0.00	Hypothetical	No similarity	hypothetical protein bll6093
bll2636	2.34	0.00	Hypothetical	Conserved hypothetical protein	hypothetical protein bll2636
blr0848	2.34	0.00	Hypothetical	Conserved hypothetical protein	hypothetical protein blr0848
bll0005	2.33	0.00	Hypothetical	No similarity	hypothetical protein bll0005
bll8237	2.33	0.00	Hypothetical	No similarity	hypothetical protein bll8237
blr1726	2.33	0.00	Hypothetical	No similarity	hypothetical protein blr1726
blr4841	2.32	0.00	Hypothetical	No similarity	hypothetical protein blr4841
blr1264	2.31	0.00	Hypothetical	No similarity	hypothetical protein blr1264
blr2370	2.31	0.00	Hypothetical	No similarity	hypothetical protein blr2370
bll0465	2.30	0.00	Hypothetical	Conserved hypothetical protein	hypothetical protein bll0465
bll4243	2.29	0.00	Hypothetical	No similarity	hypothetical protein bll4243
blr5550	2.29	0.00	Hypothetical	Conserved hypothetical protein	hypothetical protein blr5550
bll6755	2.29	0.00	Hypothetical	Conserved hypothetical protein	hypothetical protein bll6755
blr5909	2.29	0.00	Hypothetical	Conserved hypothetical protein	hypothetical protein blr5909
bsr0136	2.29	0.00	Hypothetical	Conserved hypothetical protein	hypothetical protein bsr0136
bll3088	2.29	0.00	Hypothetical	No similarity	hypothetical protein bll3088
bll5662	2.28	0.00	Hypothetical	Conserved hypothetical protein	hypothetical protein bll5662
blr7595	2.27	0.00	Hypothetical	Conserved hypothetical protein	hypothetical protein blr7595
blr5212	2.27	0.00	Hypothetical	Conserved hypothetical protein	hypothetical protein blr5212
blr7980	2.26	0.00	Hypothetical	Conserved hypothetical protein	hypothetical protein blr7980
blr1487	2.26	0.00	Hypothetical	Conserved hypothetical protein	hypothetical protein blr1487
bll0734	2.26	0.00	Hypothetical	Conserved hypothetical protein	hypothetical protein bll0734
bll3145	2.26	0.00	Hypothetical	No similarity	hypothetical protein bll3145
bll1797	2.25	0.00	Hypothetical	Conserved hypothetical protein	hypothetical protein bll1797
blr2981	2.25	0.00	Hypothetical	Conserved hypothetical protein	hypothetical protein blr2981
bll6165	2.24	0.00	Hypothetical	Conserved hypothetical protein	hypothetical protein bll6165
bll5319	2.24	0.00	Hypothetical	No similarity	hypothetical protein bll5319
bll7976	2.24	0.00	Hypothetical	Conserved hypothetical protein	hypothetical protein bll7976
blr1089	2.24	0.00	Hypothetical	Conserved hypothetical protein	hypothetical protein blr1089
bll2228	2.24	0.00	Hypothetical	No similarity	hypothetical protein bll2228
bll4786	2.24	0.00	Hypothetical	Conserved hypothetical protein	hypothetical protein bll4786
blr3063	2.23	0.00	Hypothetical	No similarity	hypothetical protein blr3063
blr3406	2.22	0.00	Hypothetical	Conserved hypothetical protein	hypothetical protein blr3406
blr5523	2.22	0.00	Hypothetical	Conserved hypothetical protein	hypothetical protein blr5523
bll5635	2.21	0.00	Hypothetical	Conserved hypothetical protein	hypothetical protein bll5635
bsr7110	2.21	0.00	Hypothetical	Conserved hypothetical protein	hypothetical protein bsr7110
blr3438	2.21	0.00	Hypothetical	Conserved hypothetical protein	hypothetical protein blr3438
blr1330.1n	2.21	0.00	Hypothetical		hypothetical protein blr1330.1n
blr0865	2.20	0.00	Hypothetical	No similarity	hypothetical protein blr0865
bll0800	2.20	0.00	Hypothetical	Conserved hypothetical protein	hypothetical protein bll0800
bll0531	2.19	0.00	Hypothetical	Conserved hypothetical protein	hypothetical protein bll0531
blr3404	2.19	0.00	Hypothetical	No similarity	hypothetical protein blr3404
blr5541	2.19	0.00	Hypothetical	Conserved hypothetical protein	hypothetical protein blr5541
bsr4969	2.18	0.00	Hypothetical	Conserved hypothetical protein	hypothetical protein bsr4969
blr1614	2.18	0.00	Hypothetical	Conserved hypothetical protein	hypothetical protein blr1614
bll7018	2.17	0.00	Hypothetical	No similarity	hypothetical protein bll7018
bll5557	2.17	0.00	Hypothetical	Conserved hypothetical protein	hypothetical protein bll5557
blr6990	2.17	0.00	Hypothetical	Conserved hypothetical protein	hypothetical protein blr6990
bll1166	2.17	0.00	Hypothetical	Conserved hypothetical protein	hypothetical protein bll1166
bll6691	2.17	0.00	Hypothetical	Conserved hypothetical protein	hypothetical protein bll6691

blr2418.2n	2.17	0.00	Hypothetical		hypothetical protein blr2418.2n
bll6730	2.16	0.00	Hypothetical	Conserved hypothetical protein	hypothetical protein bll6730
blr0101	2.16	0.00	Hypothetical	Conserved hypothetical protein	hypothetical protein blr0101
bll0776	2.16	0.00	Hypothetical	No similarity	hypothetical protein bll0776
blr5467	2.16	0.00	Hypothetical	No similarity	hypothetical protein blr5467
blr0478	2.16	0.00	Hypothetical	Conserved hypothetical protein	hypothetical protein blr0478
blr5039	2.15	0.00	Hypothetical	Conserved hypothetical protein	hypothetical protein blr5039
blr2865	2.15	0.00	Hypothetical	No similarity	hypothetical protein blr2865
blr1573	2.15	0.00	Hypothetical	Conserved hypothetical protein	hypothetical protein blr1573
blr4438	2.15	0.00	Hypothetical	Conserved hypothetical protein	hypothetical protein blr4438
blr6268	2.15	0.00	Hypothetical	Conserved hypothetical protein	hypothetical protein blr6268
blr2839	2.14	0.00	Hypothetical	Conserved hypothetical protein	hypothetical protein blr2839
bll1024	2.14	0.00	Hypothetical	Conserved hypothetical protein	hypothetical protein bll1024
bll0556	2.14	0.00	Hypothetical	Conserved hypothetical protein	hypothetical protein bll0556
bll5189	2.13	0.00	Hypothetical	Conserved hypothetical protein	hypothetical protein bll5189
bsl4665	2.13	0.00	Hypothetical	No similarity	hypothetical protein bsl4665
bll1591	2.13	0.00	Hypothetical	No similarity	hypothetical protein bll1591
bll2378	2.13	0.00	Hypothetical	No similarity	hypothetical protein bll2378
bll1109.1n	2.13	0.00	Hypothetical		hypothetical protein bll1109.1n
bsl6099	2.12	0.00	Hypothetical	No similarity	hypothetical protein bsl6099
bll5287	2.12	0.00	Hypothetical	No similarity	hypothetical protein bll5287
blr2944	2.12	0.00	Hypothetical	Conserved hypothetical protein	hypothetical protein blr2944
blr0366	2.12	0.00	Hypothetical	No similarity	hypothetical protein blr0366
blr1748	2.11	0.00	Hypothetical	Conserved hypothetical protein	hypothetical protein blr1748
bll5355	2.11	0.00	Hypothetical	No similarity	hypothetical protein bll5355
blr7561	2.11	0.00	Hypothetical	Conserved hypothetical protein	hypothetical protein blr7561
blr2083.1n	2.11	0.00	Hypothetical		hypothetical protein blr2083.1n
blr3862	2.10	0.00	Hypothetical	No similarity	hypothetical protein blr3862
bsr6683	2.10	0.00	Hypothetical	Conserved hypothetical protein	hypothetical protein bsr6683
bll4496	2.10	0.00	Hypothetical	Conserved hypothetical protein	hypothetical protein bll4496
blr0711	2.08	0.00	Hypothetical	Conserved hypothetical protein	hypothetical protein blr0711
bll1341	2.08	0.00	Hypothetical	Conserved hypothetical protein	hypothetical protein bll1341
bll5079	2.07	0.00	Hypothetical	No similarity	hypothetical protein bll5079
bll1592	2.07	0.00	Hypothetical	No similarity	hypothetical protein bll1592
bll3006	2.07	0.00	Hypothetical	Conserved hypothetical protein	hypothetical protein bll3006
bll4722	2.06	0.00	Hypothetical	Conserved hypothetical protein	hypothetical protein bll4722
bsr1809	2.06	0.00	Hypothetical	No similarity	hypothetical protein bsr1809
bll6294	2.06	0.00	Hypothetical	No similarity	hypothetical protein bll6294
bll7907	2.06	0.00	Hypothetical	Conserved hypothetical protein	hypothetical protein bll7907
bll0480	2.06	0.00	Hypothetical	Conserved hypothetical protein	hypothetical protein bll0480
blr0571	2.06	0.00	Hypothetical	Conserved hypothetical protein	hypothetical protein blr0571
blr1613	2.05	0.00	Hypothetical	Conserved hypothetical protein	hypothetical protein blr1613
bll1948	2.05	0.00	Hypothetical	No similarity	hypothetical protein bll1948
blr4344	2.05	0.00	Hypothetical	No similarity	hypothetical protein blr4344
blr3438.1n	2.05	0.00	Hypothetical		hypothetical protein blr3438.1n
bll4524	2.04	0.00	Hypothetical	Conserved hypothetical protein	hypothetical protein bll4524
blr6544	2.04	0.00	Hypothetical	No similarity	hypothetical protein blr6544
bll2637	2.04	0.00	Hypothetical	Conserved hypothetical protein	hypothetical protein bll2637
bsr4244.2n	2.04	0.00	Hypothetical		hypothetical protein bsr4244.2n
blr1269	2.03	0.00	Hypothetical	No similarity	hypothetical protein blr1269
bll3589	2.03	0.00	Hypothetical	Conserved hypothetical protein	hypothetical protein bll3589
blr0278	2.03	0.00	Hypothetical	Conserved hypothetical protein	hypothetical protein blr0278
bll8026.1n	2.03	0.00	Hypothetical		hypothetical protein bll8026.1n
blr2118	2.02	0.00	Hypothetical	No similarity	hypothetical protein blr2118
bll7558	2.02	0.00	Hypothetical	No similarity	hypothetical protein bll7558
bsr1764	2.02	0.00	Hypothetical	No similarity	hypothetical protein bsr1764
bll4718	2.01	0.00	Hypothetical	No similarity	hypothetical protein bll4718
bsl7758	2.01	0.00	Hypothetical	No similarity	hypothetical protein bsl7758
bll7751	2.01	0.00	Hypothetical	No similarity	hypothetical protein bll7751
blr3843	2.01	0.00	Hypothetical	No similarity	hypothetical protein blr3843
blr6624	2.00	0.00	Hypothetical	Conserved hypothetical protein	hypothetical protein blr6624
bsr4225	2.00	0.00	Hypothetical	No similarity	hypothetical protein bsr4225
bll7634.1n	2.00	0.00	Hypothetical		hypothetical protein bll7634.1n
bll5076	-38.52	0.00	Hypothetical	Conserved hypothetical protein	hypothetical protein bll5076
blr1676	-12.59	0.00	Hypothetical	Conserved hypothetical protein	hypothetical protein blr1676
bll2824	-12.33	0.00	Hypothetical	No similarity	hypothetical protein bll2824
bll6860	-12.14	0.00	Hypothetical	No similarity	hypothetical protein bll6860
bll6847	-11.10	0.00	Hypothetical	Conserved hypothetical protein	hypothetical protein bll6847
bll6844	-10.58	0.00	Hypothetical	No similarity	hypothetical protein bll6844
blr4934	-10.29	0.00	Hypothetical	Conserved hypothetical protein	hypothetical protein blr4934
blr6883	-10.16	0.00	Hypothetical	Conserved hypothetical protein	hypothetical protein blr6883
bll6881	-9.05	0.00	Hypothetical	Conserved hypothetical protein	hypothetical protein bll6881
blr2787	-8.58	0.00	Hypothetical	No similarity	hypothetical protein blr2787
bsl0032	-8.44	0.00	Hypothetical	No similarity	hypothetical protein bsl0032
bll6859	-8.39	0.00	Hypothetical	Conserved hypothetical protein	hypothetical protein bll6859
bll5852	-7.31	0.00	Hypothetical	Conserved hypothetical protein	hypothetical protein bll5852
bll8094	-7.10	0.00	Hypothetical	No similarity	hypothetical protein bll8094
bll0031	-6.64	0.00	Hypothetical	No similarity	hypothetical protein bll0031
blr3278	-6.58	0.00	Hypothetical	Conserved hypothetical protein	hypothetical protein blr3278
bll6567	-6.22	0.00	Hypothetical	Conserved hypothetical protein	hypothetical protein bll6567
blr5246.2n	-6.19	0.00	Hypothetical		hypothetical protein blr5246.2n
bll7982	-6.05	0.00	Hypothetical	Conserved hypothetical protein	hypothetical protein bll7982
bll6849	-5.85	0.00	Hypothetical	Conserved hypothetical protein	hypothetical protein bll6849
bll5787	-5.79	0.00	Hypothetical	Conserved hypothetical protein	hypothetical protein bll5787
bsr1677	-5.53	0.00	Hypothetical	Conserved hypothetical protein	hypothetical protein bsr1677
bsr5273	-5.41	0.00	Hypothetical	No similarity	hypothetical protein bsr5273
bll6513	-5.31	0.00	Hypothetical	Conserved hypothetical protein	hypothetical protein bll6513
bsl7391	-5.27	0.00	Hypothetical	No similarity	hypothetical protein bsl7391
bll6870	-5.26	0.00	Hypothetical	Conserved hypothetical protein	hypothetical protein bll6870
blr4260	-5.24	0.00	Hypothetical	Conserved hypothetical protein	hypothetical protein blr4260
bll1862	-5.22	0.00	Hypothetical	No similarity	hypothetical protein bll1862
blr7453	-5.15	0.00	Hypothetical	Conserved hypothetical protein	hypothetical protein blr7453
bll2012	-4.81	0.00	Hypothetical	Conserved hypothetical protein	hypothetical protein bll2012
bll6747	-4.79	0.00	Hypothetical	No similarity	hypothetical protein bll6747
bll5854	-4.79	0.00	Hypothetical	No similarity	hypothetical protein bll5854
bsr4821	-4.74	0.00	Hypothetical	No similarity	hypothetical protein bsr4821
bsr4258	-4.72	0.00	Hypothetical	Conserved hypothetical protein	hypothetical protein bsr4258
bll3115	-4.69	0.00	Hypothetical	Conserved hypothetical protein	hypothetical protein bll3115
blr4259	-4.68	0.00	Hypothetical	Conserved hypothetical protein	hypothetical protein blr4259
blr4508	-4.64	0.00	Hypothetical	No similarity	hypothetical protein blr4508
blr4114	-4.58	0.00	Hypothetical	Conserved hypothetical protein	hypothetical protein blr4114
bsl1061	-4.56	0.00	Hypothetical	No similarity	hypothetical protein bsl1061
bll2647	-4.50	0.00	Hypothetical	Conserved hypothetical protein	hypothetical protein bll2647
bsr0033	-4.46	0.00	Hypothetical	Conserved hypothetical protein	hypothetical protein bsr0033
blr7346	-4.40	0.00	Hypothetical	Conserved hypothetical protein	hypothetical protein blr7346
bll8093	-4.39	0.00	Hypothetical	No similarity	hypothetical protein bll8093
blr2472	-4.32	0.00	Hypothetical	No similarity	hypothetical protein blr2472
blr7520	-4.30	0.00	Hypothetical	Conserved hypothetical protein	hypothetical protein blr7520
blr4709	-4.28	0.00	Hypothetical	Conserved hypothetical protein	hypothetical protein blr4709

bll6863	-4.27	0.00	Hypothetical	No similarity	hypothetical protein bll6863
bll1804	-4.22	0.00	Hypothetical	No similarity	hypothetical protein bll1804
blr2290	-4.18	0.00	Hypothetical	No similarity	hypothetical protein blr2290
bll6880	-4.18	0.00	Hypothetical	No similarity	hypothetical protein bll6880
blr6996	-4.18	0.00	Hypothetical	Conserved hypothetical protein	hypothetical protein blr6996
bll5657	-4.16	0.00	Hypothetical	Conserved hypothetical protein	hypothetical protein bll5657
bll6848	-4.02	0.00	Hypothetical	Conserved hypothetical protein	hypothetical protein bll6848
blr7345	-4.00	0.00	Hypothetical	No similarity	hypothetical protein blr7345
blr7502	-3.98	0.00	Hypothetical	No similarity	hypothetical protein blr7502
bll5336	-3.98	0.00	Hypothetical	No similarity	hypothetical protein bll5336
bll6500	-3.92	0.00	Hypothetical	No similarity	hypothetical protein bll6500
blr4066	-3.92	0.00	Hypothetical	Conserved hypothetical protein	hypothetical protein blr4066
bll1097	-3.85	0.00	Hypothetical	Conserved hypothetical protein	hypothetical protein bll1097
blr4120	-3.78	0.00	Hypothetical	Conserved hypothetical protein	hypothetical protein blr4120
bll6282	-3.76	0.00	Hypothetical	Conserved hypothetical protein	hypothetical protein bll6282
bll4656	-3.75	0.00	Hypothetical	Conserved hypothetical protein	hypothetical protein bll4656
bll5506	-3.74	0.00	Hypothetical	Conserved hypothetical protein	hypothetical protein bll5506
blr5025	-3.74	0.00	Hypothetical	Conserved hypothetical protein	hypothetical protein blr5025
blr5881	-3.68	0.00	Hypothetical	No similarity	hypothetical protein blr5881
bsl7085	-3.61	0.00	Hypothetical	Conserved hypothetical protein	hypothetical protein bsl7085
blr4638	-3.61	0.00	Hypothetical	Conserved hypothetical protein	hypothetical protein blr4638
bll7787	-3.56	0.00	Hypothetical	No similarity	hypothetical protein bll7787
bll4535	-3.52	0.00	Hypothetical	Conserved hypothetical protein	hypothetical protein bll4535
bll5254	-3.51	0.00	Hypothetical	No similarity	hypothetical protein bll5254
blr2762	-3.50	0.00	Hypothetical	Conserved hypothetical protein	hypothetical protein blr2762
blr0095	-3.50	0.00	Hypothetical	Conserved hypothetical protein	hypothetical protein blr0095
bll4766	-3.46	0.00	Hypothetical	Conserved hypothetical protein	hypothetical protein bll4766
bll4271	-3.43	0.00	Hypothetical	No similarity	hypothetical protein bll4271
blr4042	-3.42	0.00	Hypothetical	Conserved hypothetical protein	hypothetical protein blr4042
blr2761	-3.42	0.00	Hypothetical	Conserved hypothetical protein	hypothetical protein blr2761
bll1877	-3.41	0.00	Hypothetical	No similarity	hypothetical protein bll1877
blr3921	-3.39	0.00	Hypothetical	Conserved hypothetical protein	hypothetical protein blr3921
bll5853	-3.38	0.00	Hypothetical	Conserved hypothetical protein	hypothetical protein bll5853
blr0497	-3.35	0.00	Hypothetical	Conserved hypothetical protein	hypothetical protein blr0497
blr6067	-3.29	0.00	Hypothetical	No similarity	hypothetical protein blr6067
bll7993	-3.28	0.00	Hypothetical	Conserved hypothetical protein	hypothetical protein bll7993
blr7088	-3.26	0.00	Hypothetical	No similarity	hypothetical protein blr7088
blr4872	-3.25	0.00	Hypothetical	No similarity	hypothetical protein blr4872
bll6706	-3.23	0.00	Hypothetical	No similarity	hypothetical protein bll6706
bll2208	-3.23	0.00	Hypothetical	Conserved hypothetical protein	hypothetical protein bll2208
blr4388	-3.22	0.00	Hypothetical	Conserved hypothetical protein	hypothetical protein blr4388
bll3657	-3.21	0.00	Hypothetical	No similarity	hypothetical protein bll3657
blr5276	-3.21	0.00	Hypothetical	Conserved hypothetical protein	hypothetical protein blr5276
blr4714	-3.20	0.00	Hypothetical	No similarity	hypothetical protein blr4714
blr2140	-3.19	0.00	Hypothetical	Conserved hypothetical protein	hypothetical protein blr2140
blr5152	-3.18	0.00	Hypothetical	Conserved hypothetical protein	hypothetical protein blr5152
blr5422	-3.16	0.00	Hypothetical	Conserved hypothetical protein	hypothetical protein blr5422
blr0871	-3.14	0.00	Hypothetical	No similarity	hypothetical protein blr0871
bsl5082	-3.14	0.00	Hypothetical	Conserved hypothetical protein	hypothetical protein bsl5082
blr7305	-3.14	0.00	Hypothetical	No similarity	hypothetical protein blr7305
blr6669	-3.11	0.00	Hypothetical	No similarity	hypothetical protein blr6669
bsr6522	-3.11	0.00	Hypothetical	Conserved hypothetical protein	hypothetical protein bsr6522
bll6956	-3.08	0.00	Hypothetical	Conserved hypothetical protein	hypothetical protein bll6956
blr0395	-3.08	0.00	Hypothetical	Conserved hypothetical protein	hypothetical protein blr0395
bsl3746	-3.08	0.00	Hypothetical	No similarity	hypothetical protein bsl3746
blr5431	-3.06	0.00	Hypothetical	Conserved hypothetical protein	hypothetical protein blr5431
bll7495	-3.04	0.00	Hypothetical	Conserved hypothetical protein	hypothetical protein bll7495
bll4367	-3.02	0.00	Hypothetical	Conserved hypothetical protein	hypothetical protein bll4367
bll4817	-3.02	0.00	Hypothetical	No similarity	hypothetical protein bll4817
bll7134	-3.00	0.00	Hypothetical	Conserved hypothetical protein	hypothetical protein bll7134
bsr3925	-3.00	0.00	Hypothetical	No similarity	hypothetical protein bsr3925
bll5846	-2.98	0.00	Hypothetical	Conserved hypothetical protein	hypothetical protein bll5846
bll7494	-2.98	0.00	Hypothetical	Conserved hypothetical protein	hypothetical protein bll7494
bll0564	-2.96	0.00	Hypothetical	Conserved hypothetical protein	hypothetical protein bll0564
bsl2560	-2.96	0.00	Hypothetical	Conserved hypothetical protein	hypothetical protein bsl2560
bll4267	-2.96	0.00	Hypothetical	No similarity	hypothetical protein bll4267
bsl7135	-2.95	0.00	Hypothetical	Conserved hypothetical protein	hypothetical protein bsl7135
bsr6066	-2.94	0.00	Hypothetical	Conserved hypothetical protein	hypothetical protein bsr6066
bll4335	-2.93	0.00	Hypothetical	Conserved hypothetical protein	hypothetical protein bll4335
blr5840	-2.92	0.00	Hypothetical	Conserved hypothetical protein	hypothetical protein blr5840
blr3207	-2.90	0.00	Hypothetical	Conserved hypothetical protein	hypothetical protein blr3207
bsr7087	-2.90	0.00	Hypothetical	No similarity	hypothetical protein bsr7087
blr5147	-2.90	0.00	Hypothetical	Conserved hypothetical protein	hypothetical protein blr5147
bll7013	-2.88	0.00	Hypothetical	Conserved hypothetical protein	hypothetical protein bll7013
blr7794	-2.87	0.00	Hypothetical	No similarity	hypothetical protein blr7794
bll4239	-2.87	0.00	Hypothetical	Conserved hypothetical protein	hypothetical protein bll4239
blr7761	-2.86	0.00	Hypothetical	Conserved hypothetical protein	hypothetical protein blr7761
bll4645	-2.85	0.00	Hypothetical	Conserved hypothetical protein	hypothetical protein bll4645
blr7478	-2.82	0.00	Hypothetical	Conserved hypothetical protein	hypothetical protein blr7478
bll5849	-2.82	0.00	Hypothetical	Conserved hypothetical protein	hypothetical protein bll5849
bll4634	-2.82	0.00	Hypothetical	Conserved hypothetical protein	hypothetical protein bll4634
bll0598	-2.81	0.00	Hypothetical	Conserved hypothetical protein	hypothetical protein bll0598
bsr5794	-2.81	0.00	Hypothetical	Conserved hypothetical protein	hypothetical protein bsr5794
bll2516	-2.80	0.00	Hypothetical	Conserved hypothetical protein	hypothetical protein bll2516
bll0332	-2.80	0.00	Hypothetical	No similarity	hypothetical protein bll0332
blr1289	-2.80	0.00	Hypothetical	Conserved hypothetical protein	hypothetical protein blr1289
bll4077	-2.79	0.00	Hypothetical	Conserved hypothetical protein	hypothetical protein bll4077
bsl4602	-2.78	0.00	Hypothetical	Conserved hypothetical protein	hypothetical protein bsl4602
bll4412	-2.78	0.00	Hypothetical	Conserved hypothetical protein	hypothetical protein bll4412
bll7309	-2.78	0.00	Hypothetical	No similarity	hypothetical protein bll7309
bll3835	-2.78	0.00	Hypothetical	Conserved hypothetical protein	hypothetical protein bll3835
bll6206	-2.77	0.00	Hypothetical	Conserved hypothetical protein	hypothetical protein bll6206
blr3124	-2.77	0.00	Hypothetical	No similarity	hypothetical protein blr3124
bll2492	-2.76	0.00	Hypothetical	Conserved hypothetical protein	hypothetical protein bll2492
blr5897	-2.76	0.00	Hypothetical	Conserved hypothetical protein	hypothetical protein blr5897
bll5501	-2.75	0.00	Hypothetical	Conserved hypothetical protein	hypothetical protein bll5501
bll5315	-2.74	0.00	Hypothetical	Conserved hypothetical protein	hypothetical protein bll5315
blr4675	-2.74	0.00	Hypothetical	No similarity	hypothetical protein blr4675
bll6069	-2.72	0.00	Hypothetical	Conserved hypothetical protein	hypothetical protein bll6069
bll7538	-2.71	0.00	Hypothetical	Conserved hypothetical protein	hypothetical protein bll7538
bsr1831	-2.70	0.00	Hypothetical	No similarity	hypothetical protein bsr1831
blr5433	-2.70	0.00	Hypothetical	Conserved hypothetical protein	hypothetical protein blr5433
blr4646	-2.70	0.00	Hypothetical	Conserved hypothetical protein	hypothetical protein blr4646
bll2549	-2.70	0.00	Hypothetical	Conserved hypothetical protein	hypothetical protein bll2549
blr5428	-2.69	0.00	Hypothetical	Conserved hypothetical protein	hypothetical protein blr5428
bll5948	-2.68	0.00	Hypothetical	Conserved hypothetical protein	hypothetical protein bll5948
bll0786	-2.68	0.00	Hypothetical	Conserved hypothetical protein	hypothetical protein bll0786
bll4269	-2.68	0.00	Hypothetical	No similarity	hypothetical protein bll4269
bll4268	-2.68	0.00	Hypothetical	No similarity	hypothetical protein bll4268

bll2087	-2.68	0.00	Hypothetical	No similarity	hypothetical protein bll2087
bll4640	-2.67	0.00	Hypothetical	Conserved hypothetical protein	hypothetical protein bll4640
blr4609	-2.67	0.00	Hypothetical	Conserved hypothetical protein	hypothetical protein blr4609
bsl4407	-2.66	0.00	Hypothetical	No similarity	hypothetical protein bsl4407
blr4240	-2.64	0.00	Hypothetical	Conserved hypothetical protein	hypothetical protein blr4240
bll4819	-2.64	0.00	Hypothetical	Conserved hypothetical protein	hypothetical protein bll4819
bll5274	-2.64	0.00	Hypothetical	Conserved hypothetical protein	hypothetical protein bll5274
bll4121	-2.63	0.00	Hypothetical	Conserved hypothetical protein	hypothetical protein bll4121
blr1149	-2.61	0.00	Hypothetical	Conserved hypothetical protein	hypothetical protein blr1149
blr7721	-2.60	0.00	Hypothetical	No similarity	hypothetical protein blr7721
bll7551	-2.60	0.01	Hypothetical	No similarity	hypothetical protein bll7551
bll1840	-2.59	0.00	Hypothetical	No similarity	hypothetical protein bll1840
blr4094	-2.59	0.00	Hypothetical	No similarity	hypothetical protein blr4094
blr4373	-2.58	0.00	Hypothetical	Conserved hypothetical protein	hypothetical protein blr4373
bll2326	-2.58	0.00	Hypothetical	Conserved hypothetical protein	hypothetical protein bll2326
blr4931	-2.58	0.00	Hypothetical	Conserved hypothetical protein	hypothetical protein blr4931
bll6525	-2.58	0.00	Hypothetical	No similarity	hypothetical protein bll6525
blr2887	-2.57	0.00	Hypothetical	Conserved hypothetical protein	hypothetical protein blr2887
blr5432	-2.56	0.00	Hypothetical	Conserved hypothetical protein	hypothetical protein blr5432
blr7061	-2.54	0.00	Hypothetical	Conserved hypothetical protein	hypothetical protein blr7061
blr7724	-2.53	0.00	Hypothetical	Conserved hypothetical protein	hypothetical protein blr7724
blr0872	-2.52	0.00	Hypothetical	No similarity	hypothetical protein blr0872
blr2611	-2.52	0.00	Hypothetical	No similarity	hypothetical protein blr2611
blr2710	-2.51	0.00	Hypothetical	No similarity	hypothetical protein blr2710
blr5470	-2.51	0.00	Hypothetical	No similarity	hypothetical protein blr5470
bll5454	-2.50	0.00	Hypothetical	Conserved hypothetical protein	hypothetical protein bll5454
blr2350	-2.49	0.00	Hypothetical	Conserved hypothetical protein	hypothetical protein blr2350
bll5845	-2.49	0.00	Hypothetical	Conserved hypothetical protein	hypothetical protein bll5845
bll4643	-2.47	0.01	Hypothetical	Conserved hypothetical protein	hypothetical protein bll4643
bll4753	-2.47	0.00	Hypothetical	Conserved hypothetical protein	hypothetical protein bll4753
bsl1808	-2.47	0.00	Hypothetical	No similarity	hypothetical protein bsl1808
blr4495	-2.46	0.00	Hypothetical	Conserved hypothetical protein	hypothetical protein blr4495
bll0224	-2.46	0.00	Hypothetical	Conserved hypothetical protein	hypothetical protein bll0224
bll5337	-2.43	0.00	Hypothetical	No similarity	hypothetical protein bll5337
bll2471	-2.42	0.00	Hypothetical	Conserved hypothetical protein	hypothetical protein bll2471
blr3164	-2.42	0.00	Hypothetical	Conserved hypothetical protein	hypothetical protein blr3164
bll7344	-2.41	0.00	Hypothetical	Conserved hypothetical protein	hypothetical protein bll7344
bll4651	-2.40	0.00	Hypothetical	Conserved hypothetical protein	hypothetical protein bll4651
blr3045	-2.39	0.00	Hypothetical	No similarity	hypothetical protein blr3045
bll2518	-2.39	0.00	Hypothetical	Conserved hypothetical protein	hypothetical protein bll2518
bsr2822	-2.37	0.00	Hypothetical	Conserved hypothetical protein	hypothetical protein bsr2822
blr5430	-2.36	0.00	Hypothetical	Conserved hypothetical protein	hypothetical protein blr5430
blr7997	-2.36	0.00	Hypothetical	No similarity	hypothetical protein blr7997
bsr7348	-2.35	0.00	Hypothetical	No similarity	hypothetical protein bsr7348
bll7406	-2.35	0.00	Hypothetical	Conserved hypothetical protein	hypothetical protein bll7406
bll6514	-2.34	0.00	Hypothetical	Conserved hypothetical protein	hypothetical protein bll6514
bll5843	-2.33	0.00	Hypothetical	Conserved hypothetical protein	hypothetical protein bll5843
bsr4406	-2.33	0.00	Hypothetical	No similarity	hypothetical protein bsr4406
bsl2070.3n	-2.33	0.00	Hypothetical		hypothetical protein bsl2070.3n
blr1534	-2.31	0.00	Hypothetical	Conserved hypothetical protein	hypothetical protein blr1534
blr2818	-2.30	0.00	Hypothetical	Conserved hypothetical protein	hypothetical protein blr2818
bll4584	-2.30	0.00	Hypothetical	Conserved hypothetical protein	hypothetical protein bll4584
bsr7384	-2.29	0.00	Hypothetical	No similarity	hypothetical protein bsr7384
bll3751	-2.29	0.00	Hypothetical	Conserved hypothetical protein	hypothetical protein bll3751
bll4036	-2.28	0.00	Hypothetical	No similarity	hypothetical protein bll4036
blr6000	-2.28	0.00	Hypothetical	No similarity	hypothetical protein blr6000
blr6562	-2.27	0.00	Hypothetical	No similarity	hypothetical protein blr6562
blr2852	-2.27	0.00	Hypothetical	Conserved hypothetical protein	hypothetical protein blr2852
blr4096	-2.26	0.00	Hypothetical	No similarity	hypothetical protein blr4096
blr2988	-2.26	0.00	Hypothetical	Conserved hypothetical protein	hypothetical protein blr2988
bll6129	-2.26	0.00	Hypothetical	Conserved hypothetical protein	hypothetical protein bll6129
bsr3779	-2.24	0.00	Hypothetical	Conserved hypothetical protein	hypothetical protein bsr3779
bsr6460	-2.24	0.00	Hypothetical	Conserved hypothetical protein	hypothetical protein bsr6460
blr7228	-2.23	0.00	Hypothetical	Conserved hypothetical protein	hypothetical protein blr7228
bll6183	-2.22	0.00	Hypothetical	Conserved hypothetical protein	hypothetical protein bll6183
bsl4647	-2.21	0.00	Hypothetical	No similarity	hypothetical protein bsl4647
bll6673	-2.21	0.00	Hypothetical	No similarity	hypothetical protein bll6673
bsr7720	-2.21	0.00	Hypothetical	No similarity	hypothetical protein bsr7720
bll5851	-2.20	0.00	Hypothetical	Conserved hypothetical protein	hypothetical protein bll5851
blr5424	-2.20	0.00	Hypothetical	Conserved hypothetical protein	hypothetical protein blr5424
bll4220	-2.20	0.00	Hypothetical	Conserved hypothetical protein	hypothetical protein bll4220
bll4691	-2.19	0.00	Hypothetical	Conserved hypothetical protein	hypothetical protein bll4691
bll1313	-2.19	0.00	Hypothetical	Conserved hypothetical protein	hypothetical protein bll1313
blr5429	-2.18	0.00	Hypothetical	Conserved hypothetical protein	hypothetical protein blr5429
bll6515	-2.18	0.00	Hypothetical	No similarity	hypothetical protein bll6515
blr4093	-2.18	0.00	Hypothetical	No similarity	hypothetical protein blr4093
bsl1652	-2.18	0.00	Hypothetical	No similarity	hypothetical protein bsl1652
blr2341	-2.17	0.00	Hypothetical	No similarity	hypothetical protein blr2341
blr5425	-2.17	0.00	Hypothetical	Conserved hypothetical protein	hypothetical protein blr5425
blr4022	-2.16	0.00	Hypothetical	No similarity	hypothetical protein blr4022
bll5946	-2.15	0.00	Hypothetical	Conserved hypothetical protein	hypothetical protein bll5946
blr1704	-2.14	0.00	Hypothetical	Conserved hypothetical protein	hypothetical protein blr1704
blr7050	-2.14	0.00	Hypothetical	No similarity	hypothetical protein blr7050
bll4788	-2.14	0.00	Hypothetical	Conserved hypothetical protein	hypothetical protein bll4788
bll6957	-2.14	0.00	Hypothetical	Conserved hypothetical protein	hypothetical protein bll6957
blr3741	-2.13	0.00	Hypothetical	Conserved hypothetical protein	hypothetical protein blr3741
blr8186	-2.13	0.00	Hypothetical	Conserved hypothetical protein	hypothetical protein blr8186
bll2618	-2.12	0.00	Hypothetical	Conserved hypothetical protein	hypothetical protein bll2618
bsl3606	-2.12	0.00	Hypothetical	No similarity	hypothetical protein bsl3606
blr6135	-2.11	0.00	Hypothetical	No similarity	hypothetical protein blr6135
blr7499	-2.11	0.00	Hypothetical	No similarity	hypothetical protein blr7499
blr4813	-2.11	0.00	Hypothetical	Conserved hypothetical protein	hypothetical protein blr4813
bll2483	-2.10	0.00	Hypothetical	Conserved hypothetical protein	hypothetical protein bll2483
bll7766	-2.10	0.00	Hypothetical	Conserved hypothetical protein	hypothetical protein bll7766
blr4262	-2.10	0.00	Hypothetical	Conserved hypothetical protein	hypothetical protein blr4262
blr4035	-2.09	0.00	Hypothetical	Conserved hypothetical protein	hypothetical protein blr4035
blr5921	-2.09	0.00	Hypothetical	Conserved hypothetical protein	hypothetical protein blr5921
blr7738	-2.09	0.00	Hypothetical	Conserved hypothetical protein	hypothetical protein blr7738
blr5994	-2.08	0.00	Hypothetical	Conserved hypothetical protein	hypothetical protein blr5994
bll5943	-2.08	0.00	Hypothetical	No similarity	hypothetical protein bll5943
bsr4097	-2.07	0.00	Hypothetical	No similarity	hypothetical protein bsr4097
blr2614	-2.07	0.00	Hypothetical	Conserved hypothetical protein	hypothetical protein blr2614
bll3113	-2.06	0.00	Hypothetical	No similarity	hypothetical protein bll3113
blr7500	-2.06	0.00	Hypothetical	No similarity	hypothetical protein blr7500
blr6628	-2.06	0.00	Hypothetical	No similarity	hypothetical protein blr6628
blr4679	-2.06	0.00	Hypothetical	Conserved hypothetical protein	hypothetical protein blr4679
bll0737	-2.05	0.00	Hypothetical	Conserved hypothetical protein	hypothetical protein bll0737
bll4765	-2.05	0.00	Hypothetical	No similarity	hypothetical protein bll4765
bll7415	-2.04	0.00	Hypothetical	Conserved hypothetical protein	hypothetical protein bll7415

bll2365	-2.04	0.00	Hypothetical	No similarity	hypothetical protein bll2365
bll5629	-2.04	0.00	Hypothetical	Conserved hypothetical protein	hypothetical protein bll5629
blr5569	-2.04	0.00	Hypothetical	Conserved hypothetical protein	hypothetical protein blr5569
bll5552	-2.03	0.00	Hypothetical	Conserved hypothetical protein	hypothetical protein bll5552
bsl2070	-2.02	0.00	Hypothetical	Conserved hypothetical protein	hypothetical protein bsl2070
blr1785	-2.02	0.00	Hypothetical	No similarity	hypothetical protein blr1785
bll4752	-2.02	0.00	Hypothetical	Conserved hypothetical protein	hypothetical protein bll4752
bsl6534	-2.02	0.00	Hypothetical	No similarity	hypothetical protein bsl6534
bll7438.1n	-2.02	0.00	Hypothetical		hypothetical protein bll7438.1n
blr1400	-2.01	0.00	Hypothetical	Conserved hypothetical protein	hypothetical protein blr1400
blr0580	-2.01	0.00	Hypothetical	Conserved hypothetical protein	hypothetical protein blr0580
bll3463	-2.01	0.00	Hypothetical	No similarity	hypothetical protein bll3463
bll6524	-2.01	0.00	Hypothetical	Conserved hypothetical protein	hypothetical protein bll6524
blr1817	-2.01	0.00	Hypothetical	Conserved hypothetical protein	hypothetical protein blr1817
blr4174	-2.01	0.00	Hypothetical	Conserved hypothetical protein	hypothetical protein blr4174
bsl3211	-2.00	0.00	Hypothetical	Conserved hypothetical protein	hypothetical protein bsl3211
blr0949	-2.00	0.00	Hypothetical	Conserved hypothetical protein	hypothetical protein blr0949
bll4334	-2.00	0.00	Hypothetical	No similarity	hypothetical protein bll4334
blr4043	-2.00	0.00	Hypothetical	Conserved hypothetical protein	hypothetical protein blr4043
bll7938	-2.00	0.00	Hypothetical	Conserved hypothetical protein	hypothetical protein bll7938
bll5844	-2.00	0.00	Hypothetical	Conserved hypothetical protein	hypothetical protein bll5844
blr3400	31.41	0.00	Other categories	Other	putative oxidoreductase
blr3401	21.59	0.00	Other categories	Other	probable aromatic 1,2-dioxygenase beta subunit
blr3412	20.83	0.00	Other categories	Other	putative gentisate 1,2-dioxygenase
blr3436	20.51	0.00	Other categories	Other	probable bifunctional 2-amninobenzoyl-CoA monooxygenase/reductase
blr3428	17.13	0.00	Other categories	Drug and analog sensitivity	organic hydroperoxide resistance protein
blr3399	16.54	0.00	Other categories	Other	vanillate O-demethylase oxidoreductase
blr3403	12.48	0.00	Other categories	Other	putative 3-oxoacyl-[acyl-carrier-protein] reductase
bll2445	11.19	0.00	Other categories	Other	oxidoreductase
bll7010	10.91	0.00	Other categories	Other	sulfonate monooxygenase
blr2178.1n	8.98	0.00	Other categories	Other	two-component hybrid sensor and regulator
blr6218	8.90	0.00	Other categories	Other	putative oxidoreductase protein
blr5045	8.67	0.00	Other categories	Other	putative inner membrane protein
blr6532	8.14	0.00	Other categories	Other	putative dehydrogenase
bll6841	8.13	0.00	Other categories	Other	putative carboxymethylenebutenolidase
bll6082	7.15	0.00	Other categories	Other	putative oxidoreductase
blr3226	5.75	0.00	Other categories	Other	ribitol kinase
bll0175	5.66	0.00	Other categories	Other	probable oxidoreductase
bll2737	5.64	0.00	Other categories	Other	oxidoreductase with iron-sulfur subunit
bll2067	5.60	0.00	Other categories	Symbiosis	nodulate formation efficiency C protein
blr3430	5.53	0.00	Other categories	Other	putative short chain dehydrogenase
blr7560	5.36	0.00	Other categories	Other	2-haloalkanoic acid dehalogenase
bll0489	5.34	0.00	Other categories	Other	putative glycine-rich protein
blr0589	5.23	0.00	Other categories	Other	probable 2-dehydropantoate 2-reductase
blr2534.1n	4.94	0.00	Other categories	Other	proteinase
bll5338	4.77	0.00	Other categories	Other	probable oxidoreductase
bll4607.1n	4.66	0.00	Other categories	Other	glucosamine-fructose-6-phosphate aminotransferase
bll7880	4.61	0.00	Other categories	Other	putative oxidoreductase protein
blr3294	4.44	0.00	Other categories	Other	putative decarboxylase
bll6451	4.35	0.00	Other categories	Other	probable alkanesulfonate monooxygenase
bll6950	4.33	0.00	Other categories	Other	putative pyrophosphorylase
blr5346	4.27	0.00	Other categories	Other	putative hydrolase
blr2606	4.23	0.00	Other categories	Other	2-nitropropane dioxygenase
blr5116	4.10	0.00	Other categories	Other	Nrd protein
blr6083	4.06	0.00	Other categories	Other	putative hydrolase
bll6092	3.97	0.00	Other categories	Other	probable dihydroxy-acid dehydratase
bll3033	3.70	0.00	Other categories	Other	probable hydrolase
bll7179	3.52	0.00	Other categories	Other	putative N-methylhydantoinase B
blr2358	3.47	0.00	Other categories	Other	probable glycosyl transferase
bll3065	3.35	0.00	Other categories	Other	hypothetical aldolase class II protein
bll7286	3.31	0.00	Other categories	Other	pyruvate oxidase
blr4870	3.28	0.00	Other categories	Drug and analog sensitivity	MFS permease
bll5373	3.13	0.00	Other categories	Other	probable short-chain dehydrogenase
blr4156	3.09	0.00	Other categories	Other	probable acetylornitine deacetylase
bll3142	2.97	0.00	Other categories	Other	putative quinone oxidoreductase
blr8188	2.97	0.00	Other categories	Other	putative dehalogenase
blr6046	2.96	0.00	Other categories	Other	putative oxidoreductase protein
bll1741	2.93	0.00	Other categories	Transposon-related functions	RSbeta~putative transposase
blr6729	2.90	0.00	Other categories	Other	putative decarboxylase
blr4186	2.89	0.00	Other categories	Drug and analog sensitivity	putative beta-lactamase
blr3831.1n	2.86	0.00	Other categories	Other	ferredoxin NADP+ reductase
bll1168	2.79	0.00	Other categories	Other	TldD protein
blr1420	2.71	0.00	Other categories	Other	flavin dependant oxidoreductase
blr0117	2.67	0.00	Other categories	Other	putative 2-dehydropantoate 2-reductase
bll7007	2.66	0.00	Other categories	Other	putative oxidoreductase
blr2861	2.66	0.00	Other categories	Drug and analog sensitivity	probable RND efflux transporter
bll1531	2.65	0.00	Other categories	Other	probable oxidoreductase
blr2073	2.64	0.00	Other categories	Symbiosis	NoeE homolog
bll3874	2.62	0.00	Other categories	Other	putative amidase
blr8306	2.59	0.00	Other categories	Transposon-related functions	putative transposase
bll0134	2.55	0.00	Other categories	Other	putative oxidoreductase
blr7589	2.55	0.00	Other categories	Other	putative oxidoreductase
bll1529	2.55	0.00	Other categories	Other	probable alcohol dehydrogenase
blr2622.1n	2.55	0.00	Other categories	Other	magnesium/cobalt transport protein
blr0899	2.52	0.00	Other categories	Other	poly(3-hydroxyalkanoate) depolymerase
blr7979	2.52	0.00	Other categories	Other	monooxygenase
bll0837	2.52	0.00	Other categories	Other	putative carboxymethylenebutenolidase
blr7422	2.49	0.00	Other categories	Other	oxidoreductase
blr4770	2.47	0.00	Other categories	Other	putative soluble lytic transglycosylase
bll4320	2.46	0.00	Other categories	Drug and analog sensitivity	probable RND efflux membrane fusion protein
bll1295	2.44	0.00	Other categories	Other	probable oxidoreductase
blr6737	2.42	0.00	Other categories	Other	probable pyrroloquinoline quinone synthesis protein C
blr1629	2.42	0.00	Other categories	Drug and analog sensitivity	multidrug resistance protein
bll0393.1n	2.41	0.00	Other categories	Other	two-component hybrid sensor and regulator
bll8163	2.39	0.00	Other categories	Other	glycosyl transferase
blr7593	2.37	0.00	Other categories	Drug and analog sensitivity	multidrug resistance efflux pump
blr6743	2.37	0.00	Other categories	Other	putative ferredoxin oxidoreductase alpha subunit
blr5937	2.36	0.00	Other categories	Other	gluconate 5-dehydrogenase
blr1827	2.36	0.00	Other categories	Transposon-related functions	putative transposase
bll4012	2.34	0.00	Other categories	Drug and analog sensitivity	organic hydroperoxide resistance protein
bll2752	2.33	0.00	Other categories	Other	probable glycosyl transferase
bll6622	2.32	0.00	Other categories	Drug and analog sensitivity	multidrug resistance efflux pump
bll1244	2.32	0.00	Other categories	Other	flavin dependant oxidoreductase
bll2692	2.30	0.00	Other categories	Other	putative glycosyltransferase
bll4952	2.29	0.00	Other categories	Symbiosis	NfeD protein homolog
bll0088	2.29	0.00	Other categories	Other	oxidoreductase
bll5898	2.26	0.00	Other categories	Other	probable dehydrogenase
bll3850	2.25	0.00	Other categories	Drug and analog sensitivity	putative multidrug resistance protein
blr6744	2.19	0.00	Other categories	Other	putative ferredoxin oxidoreductase beta subunit

bll7217	2.18	0.00	Other categories	Transposon-related functions	probable site-specific integrase/recombinase
blr7604	2.15	0.00	Other categories	Other	probable benzoylformate decarboxylase
bll5092	2.15	0.00	Other categories	Other	putative oxidoreductase
bll2376	2.15	0.00	Other categories	Other	probable glycosyl transferase
bll0179	2.13	0.00	Other categories	Other	putative acetyltransferase
bll1101	2.12	0.00	Other categories	Other	ApaG protein
blr0373	2.11	0.00	Other categories	Other	hypothetical aminotransferase
bll4319	2.11	0.00	Other categories	Drug and analog sensitivity	putative multidrag resistance protein
bll1714	2.11	0.00	Other categories	Symbiosis	two component regulator
blr2578	2.10	0.00	Other categories	Drug and analog sensitivity	MFS permease
blr1971	2.08	0.00	Other categories	Other	putative peptidase
blr6725	2.08	0.00	Other categories	Drug and analog sensitivity	putative multidrug resistance protein
blr6084	2.06	0.00	Other categories	Other	BEC protein
bll7407	2.04	0.00	Other categories	Drug and analog sensitivity	fosmidomycin resistance protein
blr0463	2.03	0.00	Other categories	Other	acetyltransferase
blr8233	2.03	0.00	Other categories	Transposon-related functions	putative transposase
blr5594	2.03	0.00	Other categories	Drug and analog sensitivity	MFS permease
bll1317	2.03	0.00	Other categories	Other	peroxiredoxin
blr1721	2.03	0.00	Other categories	Hydrogenase	uptake hydrogenase large subunit homolog
bll2329	2.00	0.00	Other categories	Other	putative monooxygenase
bsr8214	2.00	0.00	Other categories	Transposon-related functions	putative transposase
blr5658	-13.59	0.00	Other categories	Other	putative avidin
bll7981	-5.65	0.00	Other categories	Other	putative dehydrogenase
blr1812	-4.85	0.00	Other categories	Symbiosis	nodulation protein
blr2025	-4.78	0.00	Other categories	Symbiosis	acyl transferase
bll0333	-4.72	0.00	Other categories	Other	probable alcohol dehydrogenase precursor
bll4252	-4.25	0.00	Other categories	Other	putative hydrolase
bll3116	-3.58	0.00	Other categories	Other	putative phosphoribosylpyrophosphate synthetase
blr1993	-3.43	0.00	Other categories	Other	probable polygalacturonase
blr5623	-3.43	0.00	Other categories	Other	TPR domain protein
blr5427	-3.39	0.00	Other categories	Other	hypothetical transketolase family protein
bll2209	-3.32	0.00	Other categories	Adaptations and atypical conditions	copper tolerance protein
bll6175	-3.28	0.00	Other categories	Other	probable DNA-binding protein HU 1
bll2211	-3.24	0.00	Other categories	Adaptations and atypical conditions	copper tolerance protein
blr3205	-3.10	0.00	Other categories	Other	dehydrogenase
blr5421	-3.07	0.00	Other categories	Other	dehydratase-like protein
blr0034	-3.03	0.00	Other categories	Transposon-related functions	putative transposase
bll5848	-2.95	0.00	Other categories	Other	putative decarboxylase
blr1815	-2.89	0.00	Other categories	Symbiosis	nodulation protein
blr4083	-2.88	0.00	Other categories	Other	3-oxoacyl-(acyl carrier protein) reductase
bll5655	-2.87	0.00	Other categories	Other	alcohol dehydrogenase
blr5278	-2.80	0.00	Other categories	Other	IdnD L-idonate 5-dehydrogenase
blr5426	-2.80	0.00	Other categories	Other	transketolase
bll6831	-2.78	0.00	Other categories	Other	L-idonate 5-dehydrogenase
blr5277	-2.78	0.00	Other categories	Other	gluconate dehydrogenase
bll4389	-2.67	0.00	Other categories	Other	phospho-2-dehydro-3-deoxyheptonate aldolase
bll3117	-2.67	0.00	Other categories	Other	putative thymidine phosphorylase
blr6677	-2.66	0.00	Other categories	Other	putative hydrolase
bll0225	-2.65	0.00	Other categories	Other	acetoacetyl CoA reductase
blr2898	-2.62	0.00	Other categories	Hydrogenase	putative zinc binding dehydrogenase
bll5927	-2.60	0.00	Other categories	Other	probable ADP-heptose synthase
blr1814	-2.57	0.00	Other categories	Symbiosis	nodulation protein
blr5972	-2.54	0.00	Other categories	Other	probable acylneuraminate cytidylyltransferase
blr5742	-2.46	0.00	Other categories	Other	putative quinone oxidoreductase
blr1994	-2.45	0.00	Other categories	Other	probable pectinesterase
bll2294	-2.44	0.00	Other categories	Other	putative alkaline protease
blr2348	-2.42	0.00	Other categories	Other	protein-glutamate O-methyltransferase
blr3742	-2.38	0.00	Other categories	Drug and analog sensitivity	probable multidrug-resistance related protein
bll4669	-2.36	0.00	Other categories	Other	putative mutator protein
bll6502	-2.32	0.00	Other categories	Other	putative amino-acid dehydratase
bll2236	-2.32	0.00	Other categories	Other	putative 4'-phosphopantetheinyl transferase
blr5975	-2.32	0.00	Other categories	Other	putative oxidoreductase
bll3913	-2.21	0.00	Other categories	Other	3-oxoacyl-(acyl-carrier protein) reductase
blr1495	-2.13	0.00	Other categories	Other	oxidoreductase
blr7289	-2.12	0.00	Other categories	Other	putative alkanal monooxygenase
bll0766	-2.12	0.00	Other categories	Other	probable dehydrogenase
bll2482	-2.08	0.00	Other categories	Other	putative 6-pyruvoyl tetrahydrobiopterin synthase
blr5976	-2.07	0.00	Other categories	Other	putative oxidoreductase
bll5917	-2.07	0.00	Other categories	Other	DegT/DnrJ/EryC1/StrS family protein
bsl8242	-2.07	0.00	Other categories	Transposon-related functions	putative transposase
bll1685	-2.05	0.00	Other categories	Transposon-related functions	putative transposase
bll2817	-2.05	0.00	Other categories	Other	gluconolactonase precursor
blr0216	-2.05	0.00	Other categories	Other	aspartokinase
bll3020	-2.03	0.00	Other categories	Other	GMC type oxidoreductase
bll6994	-2.01	0.00	Other categories	Other	putative phosphatidylethanolamine N-methyltransferase
blr2421	-2.00	0.00	Other categories	Drug and analog sensitivity	MFS permease
blr7332	2.34	0.00	Purines, pyrimidines, nucleosides, and nucleotides	Purine ribonucleotide biosynthesis	putative inosine-5'-monophosphate dehydrogenase protein
blr4119	-4.41	0.00	Purines, pyrimidines, nucleosides, and nucleotides	Interconversions and salvage of nucleosides and nucleotides	nucleoside diphosphate kinase
bll4274	-3.88	0.00	Purines, pyrimidines, nucleosides, and nucleotides	Purine ribonucleotide biosynthesis	ribonucleoside-diphosphate reductase 2 alpha chain
blr0581	-3.75	0.00	Purines, pyrimidines, nucleosides, and nucleotides	Purine ribonucleotide biosynthesis	bifunctional purine biosynthesis protein
bll4859	-3.62	0.00	Purines, pyrimidines, nucleosides, and nucleotides	Pyrimidine ribonucleotide biosynthesis	uridylate kinase
blr4126	-2.91	0.00	Purines, pyrimidines, nucleosides, and nucleotides	Purine ribonucleotide biosynthesis	5'-phosphoribosylglycinamide formyltransferase
blr2555	-2.74	0.00	Purines, pyrimidines, nucleosides, and nucleotides	Pyrimidine ribonucleotide biosynthesis	dihydroorotase
bll7498	-2.73	0.00	Purines, pyrimidines, nucleosides, and nucleotides	Purine ribonucleotide biosynthesis	phosphoribosylamine--glycine ligase
bll5955	-2.61	0.00	Purines, pyrimidines, nucleosides, and nucleotides	Purine ribonucleotide biosynthesis	formyltetrahydrofolate deformylase
blr7377	-2.52	0.00	Purines, pyrimidines, nucleosides, and nucleotides	Pyrimidine ribonucleotide biosynthesis	carbamoylphosphate synthase large subunit
bll7052	-2.29	0.00	Purines, pyrimidines, nucleosides, and nucleotides	Purine ribonucleotide biosynthesis	adenylosuccinate synthetase
bll5719	-2.25	0.00	Purines, pyrimidines, nucleosides, and nucleotides	Purine ribonucleotide biosynthesis	phosphoribosylformylglycinamidine synthetase II
blr7115	-2.21	0.00	Purines, pyrimidines, nucleosides, and nucleotides	Purine ribonucleotide biosynthesis	phosphoribosylaminoimidazole carboxylase ATPase subunit
blr4125	-2.19	0.00	Purines, pyrimidines, nucleosides, and nucleotides	Purine ribonucleotide biosynthesis	5'-phosphoribosyl-5-aminoimidazole synthetase
bll6512	-2.10	0.00	Purines, pyrimidines, nucleosides, and nucleotides	Pyrimidine ribonucleotide biosynthesis	thymidylate synthase
blr0744	-2.08	0.00	Purines, pyrimidines, nucleosides, and nucleotides	Purine ribonucleotide biosynthesis	N-(5'-phosphoribosyl) anthranilate isomerase
blr7256	-2.01	0.00	Purines, pyrimidines, nucleosides, and nucleotides	Purine ribonucleotide biosynthesis	putative adenylate cyclase
blr2424	9.63	0.00	Regulatory functions		transcriptional regulatory protein
bll3434	8.90	0.00	Regulatory functions		transcriptional regulatory protein
bll3297	6.76	0.00	Regulatory functions		putative monoamine oxidase regulatory protein
bll2786	4.04	0.00	Regulatory functions		transcriptional regulatory protein
blr2890	3.97	0.00	Regulatory functions		phenylacetic acid degradation operon negative regulatory protein
bll2785	3.92	0.00	Regulatory functions		probable transcriptional regulator
blr2859	3.82	0.00	Regulatory functions		transcriptional regulatory protein
bll0121	3.25	0.00	Regulatory functions		transcriptional regulatory protein
blr1896	3.20	0.00	Regulatory functions		transcriptional regulatory protein
blr0299	3.19	0.00	Regulatory functions		transcriptional regulator
bll0687	3.14	0.00	Regulatory functions		transcriptional regulatory protein
bsr6672	2.97	0.00	Regulatory functions		transcriptional regulatory protein
bll2465	2.92	0.00	Regulatory functions		MoxR family protein
bll3409	2.86	0.00	Regulatory functions		transcriptional regulatory protein
bll3090	2.83	0.00	Regulatory functions		transcriptional regulatory protein
bll4033	2.78	0.00	Regulatory functions		transcriptional regulatory protein

blr5497	2.69	0.00	Regulatory functions		transcriptional regulatory protein
bll6252	2.53	0.00	Regulatory functions		transcriptional regulatory protein
bll5070	2.50	0.00	Regulatory functions		tmRNA-binding protein
blr0736	2.50	0.00	Regulatory functions		transcriptional regulatory protein
bsl4167	2.47	0.00	Regulatory functions		putative glutamine synthetase translation inhibitor
bll3408	2.41	0.00	Regulatory functions		transcriptional regulatory protein
bll2727	2.30	0.00	Regulatory functions		transcriptional regulatory protein
bll0304	2.29	0.00	Regulatory functions		two-component response regulator
bll0303	2.29	0.00	Regulatory functions		two-component sensor histidine kinase
blr2577	2.23	0.00	Regulatory functions		transcriptional regulatory protein
bsl1713	2.20	0.00	Regulatory functions		two-component response regulator
bll1112	2.18	0.00	Regulatory functions		transcriptional regulatory protein
bll0399	2.12	0.00	Regulatory functions		transcriptional regulatory protein
bll4551	2.12	0.00	Regulatory functions		two-component response regulator
blr7881	2.09	0.00	Regulatory functions		transcriptional regulatory protein
bll7081	2.08	0.00	Regulatory functions		two-component response regulator
blr8062	2.08	0.00	Regulatory functions		two-component response regulator
blr2444	2.07	0.00	Regulatory functions		transcriptional regulatory protein
blr3873	2.07	0.00	Regulatory functions		transcriptional regulatory protein
blr2862	2.06	0.00	Regulatory functions		two-component hybrid sensor and regulator
blr2697	2.02	0.00	Regulatory functions		VirA-like protein
blr4427	-7.52	0.00	Regulatory functions		transcriptional regulatory protein
blr6846	-6.54	0.00	Regulatory functions		two-component response regulator
blr2347	-4.14	0.00	Regulatory functions		putative sensory transducer protein
bll0933	-3.54	0.00	Regulatory functions		transcriptional regulatory protein
blr4599	-3.46	0.00	Regulatory functions		transcriptional regulatory protein
bll0635	-3.43	0.00	Regulatory functions		transcription termination factor
bll5275	-3.40	0.00	Regulatory functions		transcriptional regulatory protein
bll4785	-3.34	0.00	Regulatory functions		transcriptional regulatory protein
bll2109	-3.27	0.00	Regulatory functions		transcriptional regulatory protein
blr7084	-2.98	0.00	Regulatory functions		transcriptional regulatory protein
blr6272	-2.96	0.00	Regulatory functions		transcriptional regulatory protein
blr7666	-2.92	0.00	Regulatory functions		transcriptional regulatory protein
bll5683	-2.86	0.00	Regulatory functions		probable serine/threonine phosphatase
blr0540	-2.70	0.00	Regulatory functions		GTP-binding tyrosin phosphorylated protein
bll7696	-2.62	0.00	Regulatory functions		transcriptional regulatory protein
blr6616	-2.56	0.00	Regulatory functions		transcriptional regulatory protein
blr3204	-2.43	0.00	Regulatory functions		transcriptional regulatory protein
bll5808	-2.40	0.00	Regulatory functions		two-component hybrid sensor and regulator
blr6886	-2.31	0.00	Regulatory functions		transcriptional regulatory protein
blr6798	-2.24	0.00	Regulatory functions		transcriptional regulatory protein
bll0330	-2.18	0.00	Regulatory functions		two-component response regulator
bll5682	-2.13	0.00	Regulatory functions		probable serine/threonine kinase
blr0511	-2.12	0.00	Regulatory functions		transcriptional regulatory protein
bll1028	2.44	0.00	Transcription	RNA synthesis, modification, and DNA transcription	RNA polymerase sigma factor
blr1883	-3.78	0.00	Transcription	RNA synthesis, modification, and DNA transcription	RNA polymerase sigma-54 subunit
bll5376	-3.61	0.00	Transcription	RNA synthesis, modification, and DNA transcription	DNA-directed RNA polymerase alpha subunit
bll1447	-2.81	0.00	Transcription	RNA synthesis, modification, and DNA transcription	dead-box ATP-dependent RNA helicase
bll0779	-2.04	0.00	Transcription	RNA synthesis, modification, and DNA transcription	polyribonucleotide nucleotidyltransferase
bll5416	-2.00	0.00	Transcription	RNA synthesis, modification, and DNA transcription	transcription antitermination protein
blr2721	5.37	0.00	Translation	Degradation of proteins, peptides, and glycopeptides	probable serine protease do-like precursor
bll5153	4.86	0.00	Translation	Degradation of proteins, peptides, and glycopeptides	ClpA-like protein
bll7941	3.42	0.00	Translation	Degradation of proteins, peptides, and glycopeptides	aminopeptidase
blr7484	3.28	0.00	Translation	Degradation of proteins, peptides, and glycopeptides	hypothetical zinc protease
blr4308	3.02	0.00	Translation	Protein modification and translation factors	peptide chain release factor 2
blr0376	2.93	0.00	Translation	Degradation of proteins, peptides, and glycopeptides	protease II
blr5597	2.78	0.00	Translation	Degradation of proteins, peptides, and glycopeptides	putative carboxypeptidase precursor
blr7043	2.73	0.00	Translation	Protein modification and translation factors	peptide methionine sulfoxide reductase
blr6174	2.53	0.00	Translation	Degradation of proteins, peptides, and glycopeptides	ATP-dependent protease LA
blr7044	2.41	0.00	Translation	Protein modification and translation factors	peptide methionine sulfoxide reductase
blr7485	2.34	0.00	Translation	Degradation of proteins, peptides, and glycopeptides	hypothetical zinc protease
blr2960	2.09	0.00	Translation	Aminoacyl tRNA synthetases and tRNA modification	glutamyl-tRNA(Gln) amidotransferase subunit
bll4822	2.05	0.00	Translation	Degradation of proteins, peptides, and glycopeptides	carboxy-terminal protease
bll1795	2.05	0.00	Translation	Degradation of proteins, peptides, and glycopeptides	similar to zinc protease
bll5405	-5.46	0.00	Translation	Ribosomal proteins	30S ribosomal protein S12
bll4962	-5.09	0.00	Translation	Ribosomal proteins	30S ribosomal protein S9
bll5412	-4.97	0.00	Translation	Ribosomal proteins	50S ribosomal Protein L10
bll5395	-4.95	0.00	Translation	Ribosomal proteins	50S ribosomal protein L22
bll5399	-4.93	0.00	Translation	Ribosomal proteins	50S ribosomal protein L4
bll5400	-4.69	0.00	Translation	Ribosomal proteins	50S ribosomal protein L3
bll5375	-4.65	0.00	Translation	Ribosomal proteins	50S ribosomal protein L17
blr4404	-4.64	0.00	Translation	Nucleoproteins	histone-like protein
bll5383	-4.56	0.00	Translation	Ribosomal proteins	30s ribosomal protein S5
bll5389	-4.56	0.00	Translation	Ribosomal proteins	50S ribosomal protein L24
bll5390	-4.54	0.00	Translation	Ribosomal proteins	50S ribosomal protein L14
bll5386	-4.42	0.00	Translation	Ribosomal proteins	30S ribosomal protein S8
blr0420	-4.35	0.00	Translation	Ribosomal proteins	50S ribosomal protein L21
bll5398	-4.29	0.00	Translation	Ribosomal proteins	50S ribosomal protein L23
bll5746	-4.28	0.00	Translation	Aminoacyl tRNA synthetases and tRNA modification	probable tRNA/rRNA methyltransferase
bll4963	-4.18	0.00	Translation	Ribosomal proteins	50S ribosomal protein L13
bll4861	-4.18	0.00	Translation	Ribosomal proteins	30S ribosomal protein S2
bll4076	-4.03	0.00	Translation	Ribosomal proteins	50S ribosomal protein L9
bll4079	-4.02	0.00	Translation	Ribosomal proteins	30S ribosomal protein S6
bsl4078	-3.93	0.00	Translation	Ribosomal proteins	30S ribosomal protein S18
bll5403	-3.92	0.00	Translation	Protein modification and translation factors	translation elongation factor G
bll5387	-3.92	0.00	Translation	Ribosomal proteins	30S ribosomal protein S14
bll5381	-3.89	0.00	Translation	Ribosomal proteins	50S ribosomal protein L15
bll0707	-3.88	0.00	Translation	Ribosomal proteins	50S ribosomal protein L20
bll5385	-3.84	0.00	Translation	Ribosomal proteins	50S ribosomal protein L6
bll5394	-3.83	0.00	Translation	Ribosomal proteins	30S ribosomal protein S3
bsl5391	-3.81	0.00	Translation	Ribosomal proteins	30S ribosomal protein S17
bll5378	-3.73	0.00	Translation	Ribosomal proteins	30S ribosomal protein S13
bsl5396	-3.72	0.00	Translation	Ribosomal proteins	30S ribosomal protein S19
bll5393	-3.71	0.00	Translation	Ribosomal proteins	50S ribosomal protein L16
blr0483	-3.69	0.00	Translation	Ribosomal proteins	16S rRNA processing protein
blr5753	-3.66	0.00	Translation	Protein modification and translation factors	glycine cleavage system protein P2
bsl5392	-3.56	0.00	Translation	Ribosomal proteins	50S ribosomal protein L29
bll5411	-3.54	0.00	Translation	Ribosomal proteins	50S ribosomal protein L7/L12
bsl0832	-3.47	0.00	Translation	Ribosomal proteins	30S ribosomal protein S20
bll5397	-3.46	0.00	Translation	Ribosomal proteins	50S ribosomal protein L2
blr5706	-3.44	0.00	Translation	Ribosomal proteins	30S ribosomal protein S4
blr0740	-3.36	0.00	Translation	Ribosomal proteins	30S ribosomal protein S1
blr5751	-3.33	0.00	Translation	Protein modification and translation factors	glycine cleavage system component T
bll5401	-3.31	0.00	Translation	Ribosomal proteins	30S ribosomal protein S10
bll4860	-3.28	0.00	Translation	Protein modification and translation factors	translation elongation factor Ts
bll5404	-3.27	0.00	Translation	Ribosomal proteins	30S ribosomal protein S7
bll5414	-3.26	0.00	Translation	Ribosomal proteins	50S ribosomal protein L1
bll5377	-3.23	0.00	Translation	Ribosomal proteins	30S ribosomal protein S11
blr5752	-3.17	0.00	Translation	Protein modification and translation factors	glycine cleavage system component H

blr0482	-3.16	0.00	Translation	Ribosomal proteins	30S ribosomal protein S16
blr0162	-3.14	0.00	Translation	Ribosomal proteins	50S ribosomal protein L28
bll5388	-3.07	0.00	Translation	Ribosomal proteins	50S ribosomal protein L5
bll4386	-3.04	0.00	Translation	Protein modification and translation factors	elongation factor P
bll5402	-2.95	0.00	Translation	Protein modification and translation factors	elongation factor TU
bsl0780	-2.90	0.00	Translation	Ribosomal proteins	30S ribosomal protein S15
blr0487	-2.85	0.00	Translation	Ribosomal proteins	50S ribosomal protein L19
bsl5382	-2.79	0.00	Translation	Ribosomal proteins	50S ribosomal protein L30
bsl0708	-2.62	0.00	Translation	Ribosomal proteins	50S ribosomal protein L35
bll7441	-2.59	0.00	Translation	Ribosomal proteins	50S ribosomal protein L25
blr4143	-2.58	0.00	Translation	Aminoacyl tRNA synthetases and tRNA modification	aspartyl-tRNA synthetase
bll4639	-2.52	0.00	Translation	Degradation of proteins, peptides, and glycopeptides	probable protease
bll7440	-2.49	0.00	Translation	Aminoacyl tRNA synthetases and tRNA modification	peptidyl-tRNA hydrolase
bll4837	-2.43	0.00	Translation	Aminoacyl tRNA synthetases and tRNA modification	glutaminyl-tRNA synthetase
blr6588	-2.36	0.00	Translation	Ribosomal proteins	ribosomal protein L11 methyltransferase
bsr5117	-2.32	0.00	Translation	Ribosomal proteins	50S ribosomal protein L33
bll3749	-2.26	0.00	Translation	Degradation of proteins, peptides, and glycopeptides	probable carboxypeptidase G2 precursor
bll0849	-2.26	0.00	Translation	Degradation of proteins, peptides, and glycopeptides	putative serine protease
bll5415	-2.22	0.00	Translation	Ribosomal proteins	50S ribosomal Protein L11
bll4748	-2.20	0.00	Translation	Aminoacyl tRNA synthetases and tRNA modification	seryl-tRNA synthetase
bll0802	-2.17	0.00	Translation	Aminoacyl tRNA synthetases and tRNA modification	tryptophan-tRNA ligase
bll5384	-2.17	0.00	Translation	Ribosomal proteins	50S ribosomal protein L18
blr0365	-2.15	0.00	Translation	Ribosomal proteins	30S ribosomal protein S21
bll0781	-2.12	0.00	Translation	Aminoacyl tRNA synthetases and tRNA modification	tRNA pseudouridine 55 synthase
blr7524	-2.10	0.00	Translation	Aminoacyl tRNA synthetases and tRNA modification	probable ATP phosphoribosyltransferase regulatory subunit
bll5912	-2.09	0.00	Translation	Aminoacyl tRNA synthetases and tRNA modification	serine hydroxymethyltransferase
bll4330	-2.08	0.00	Translation	Aminoacyl tRNA synthetases and tRNA modification	tyrosyl-tRNA synthetase
bll6031	-2.07	0.00	Translation	Degradation of proteins, peptides, and glycopeptides	putative zinc metalloendopeptidase
bsl7548	-2.06	0.00	Translation	Ribosomal proteins	50S ribosomal protein L36
blr1133	-2.06	0.00	Translation	Aminoacyl tRNA synthetases and tRNA modification	lysyl-tRNA synthetase
bsr0948	-2.04	0.00	Translation	Ribosomal proteins	50S ribosomal protein L32
bll5087	-2.02	0.00	Translation	Aminoacyl tRNA synthetases and tRNA modification	glutamyl-tRNA amidotransferase subunit B
bll3426	50.11	0.00	Transport and binding proteins		ABC transporter substrate-binding protein
bll7011	21.47	0.00	Transport and binding proteins		ABC transporter aliphatic-sulfonate-binding protein
blr2422	18.24	0.00	Transport and binding proteins		putative efflux protein
blr3432	9.65	0.00	Transport and binding proteins		putative branched-chain amino acid transporter
bll6455	9.20	0.00	Transport and binding proteins		ABC transporter substrate-binding protein
bll4895	8.57	0.00	Transport and binding proteins		ABC transporter permease protein
bll3425	6.99	0.00	Transport and binding proteins		ABC transporter permease protein
bll7009	5.51	0.00	Transport and binding proteins		aliphatic sulfonate ABC transporter permease protein
bll7008	5.33	0.00	Transport and binding proteins		aliphatic sulfonate ABC transporter ATP-binding protein
blr2423	4.60	0.00	Transport and binding proteins		cation efflux system protein
blr6456	4.57	0.00	Transport and binding proteins		probable aliphatic sulfonates binding protein
blr3283	3.29	0.00	Transport and binding proteins		hypothetical transthyretin-like protein
blr3104	3.23	0.00	Transport and binding proteins		major facilitator superfamily transporter
blr7376	3.16	0.00	Transport and binding proteins		putative integral membrane transporter protein
blr2934	3.08	0.00	Transport and binding proteins		probable cation efflux protein
blr7187	3.02	0.00	Transport and binding proteins		C4-dicarboxylate transport protein
bll7103	2.95	0.00	Transport and binding proteins		ABC transporter permease protein
blr6078	2.65	0.00	Transport and binding proteins		probable substrate-binding protein
blr3396	2.62	0.00	Transport and binding proteins		major facilitator superfamily transporter
bll4878	2.55	0.00	Transport and binding proteins		possible Copper export protein
bll0301	2.52	0.00	Transport and binding proteins		cation efflux protein
blr6644	2.37	0.00	Transport and binding proteins		ABC transporter ATP-binding protein
blr6645	2.33	0.00	Transport and binding proteins		ABC transporter substrate-binding protein
blr1482	2.30	0.00	Transport and binding proteins		ABC transporter sulfate-binding protein
bll6232	2.27	0.00	Transport and binding proteins		ABC transporter ATP-binding protein
blr3670	2.13	0.00	Transport and binding proteins		probable substrate-binding protein
bll6551	2.10	0.00	Transport and binding proteins		probable ABC transporter substrate-binding protein
blr1352	2.04	0.00	Transport and binding proteins		aminobenzoyl-glutamate utilization protein
bll6834	-16.28	0.00	Transport and binding proteins		probable ABC transporter substrate-binding protein
blr3208	-7.76	0.00	Transport and binding proteins		ABC transporter sugar-binding protein
bll4057	-7.09	0.00	Transport and binding proteins		probable substrate-binding protein
bll6833	-6.90	0.00	Transport and binding proteins		probable ABC transporter permease protein
blr3918	-6.61	0.00	Transport and binding proteins		ABC transporter substrate-binding protein
blr3920	-5.77	0.00	Transport and binding proteins		ABC transporter permease protein
blr3200	-5.46	0.00	Transport and binding proteins		ABC transporter sugar-binding protein
bll6832	-4.99	0.00	Transport and binding proteins		probable ABC transporter permease protein
blr3743	-4.92	0.00	Transport and binding proteins		periplasmic mannitol-binding protein
blr7037	-4.11	0.00	Transport and binding proteins		periplasmic nitrate reductase
blr3917	-4.11	0.00	Transport and binding proteins		ABC transporter ATP-binding protein
blr3919	-3.99	0.00	Transport and binding proteins		ABC transporter permease protein
blr7873	-3.98	0.00	Transport and binding proteins		ABC transporter ATP-binding protein
blr3210	-3.92	0.00	Transport and binding proteins		sugar ABC transporter permease protein
blr4039	-3.87	0.00	Transport and binding proteins		ABC transporter substrate-binding protein
bll6803	-3.68	0.00	Transport and binding proteins		hypothetical metabolite transport protein
bll5951	-3.67	0.00	Transport and binding proteins		ABC transporter permease protein
blr7099	-3.66	0.00	Transport and binding proteins		probable ABC transporter substrate-binding protein
bll0733	-3.63	0.00	Transport and binding proteins		ABC transporter glycerol-3-phosphate-binding protein
blr4936	-3.56	0.00	Transport and binding proteins		putative cation efflux system protein
blr3201	-3.54	0.00	Transport and binding proteins		sugar ABC transporter ATP-binding protein
blr4115	-3.48	0.00	Transport and binding proteins		putative symporter
bll5953	-3.42	0.00	Transport and binding proteins		ABC transporter substrate-binding protein
blr4938	-3.36	0.00	Transport and binding proteins		putative cation efflux system protein
bll5496	-3.35	0.00	Transport and binding proteins		hypothetical metabolite transport protein
blr7827	-3.33	0.00	Transport and binding proteins		ABC transporter substrate-binding protein
blr7053	-3.27	0.00	Transport and binding proteins		probable cation-transporting ATPase
bll5950	-3.26	0.00	Transport and binding proteins		ABC transporter ATP-binding protein
blr7826	-3.24	0.00	Transport and binding proteins		ABC transporter permease protein
bll7988	-3.24	0.00	Transport and binding proteins		probable ATP-binding protein
blr3203	-3.21	0.00	Transport and binding proteins		sugar ABC transporter permease protein
blr4037	-3.20	0.00	Transport and binding proteins		ABC transporter ATP-binding protein
blr7825	-3.19	0.00	Transport and binding proteins		ABC transporter permease protein
bll1057	-3.08	0.00	Transport and binding proteins		ABC transporter permease protein
blr4040	-3.07	0.00	Transport and binding proteins		ABC transporter permease protein
blr3815	-3.06	0.00	Transport and binding proteins		putative cation-transporting ATPase
blr3202	-3.03	0.00	Transport and binding proteins		sugar ABC transporter permease protein
blr4511	-3.00	0.00	Transport and binding proteins		probable ABC transporter substrate-binding protein
blr7101	-2.97	0.00	Transport and binding proteins		probable ABC transporter permease protein
bll1193	-2.94	0.00	Transport and binding proteins		integral inner membrane metabolite transport protein
blr1679	-2.92	0.00	Transport and binding proteins		similar to ABC transporter permease protein
blr3209	-2.89	0.00	Transport and binding proteins		sugar ABC transporter ATP-binding protein
blr4041	-2.76	0.00	Transport and binding proteins		ABC transporter permease protein
bll2018	-2.65	0.00	Transport and binding proteins		similar to ABC transporter, amino acid binding protein
blr7100	-2.64	0.00	Transport and binding proteins		probable ABC transporter permease protein
bll6434	-2.64	0.00	Transport and binding proteins		C4-dicarboxylate-binding protein
blr7038	-2.60	0.00	Transport and binding proteins		periplasmic nitrate reductase large subunit precursor
bsr7036	-2.52	0.00	Transport and binding proteins		periplasmic nitrate reductase protein
bll5890	-2.51	0.00	Transport and binding proteins		monocarboxylic acid permease

blr5671	-2.39	0.00	Transport and binding proteins	ABC transporter permease protein
blr4298	-2.39	0.00	Transport and binding proteins	C4-dicarboxylate transport protein
blr3032	-2.37	0.00	Transport and binding proteins	cation efflux system protein
bsr4636	-2.35	0.00	Transport and binding proteins	putative cation transport regulator
blr7824	-2.35	0.00	Transport and binding proteins	ABC transporter ATP-binding protein
bll5028	-2.32	0.00	Transport and binding proteins	N-utilization substance protein B
bll0885	-2.30	0.00	Transport and binding proteins	ABC transporter ATP-binding protein
blr4112	-2.14	0.00	Transport and binding proteins	Probale cation efflux system protein
blr0607	-2.14	0.00	Transport and binding proteins	similar to ammonium transporter
bll7600	-2.13	0.00	Transport and binding proteins	ABC transporter amino-acid-binding protein
bll5044	-2.12	0.00	Transport and binding proteins	manganese transport protein
blr4500	-2.10	0.00	Transport and binding proteins	probable ABC transporter permease protein
bll7104	-2.10	0.00	Transport and binding proteins	ABC transporter permease protein
bll5949	-2.09	0.00	Transport and binding proteins	ABC transporter ATP-binding protein
bll0785	-2.08	0.00	Transport and binding proteins	N-utilization substance protein A
blr5962	-2.08	0.00	Transport and binding proteins	ABC transporter ATP-binding protein
blr4845	-2.07	0.00	Transport and binding proteins	ABC transporter ATP-binding protein
blr5970	-2.05	0.00	Transport and binding proteins	putative sugar kinase
bll0989	-2.04	0.00	Transport and binding proteins	peptide ABC transporter ATP-binding protein
bll1058	-2.00	0.00	Transport and binding proteins	ABC transporter substrate-binding protein
bll4118	-2.00	0.00	Transport and binding proteins	probable ATP-binding protein
